# Effectiveness of the implementation project ‘Don’t forget the mouth!’ of community dwelling older people with dementia: a prospective longitudinal single-blind multicentre study protocol (DFTM!)

**DOI:** 10.1186/s12903-019-0786-5

**Published:** 2019-05-28

**Authors:** Bach Van Ho, Roxane Anthea Francesca Weijenberg, Claar Debora van der Maarel-Wierink, Corine Mirjam Visscher, Gert-Jan van der Putten, Erik Johan Anton Scherder, Frank Lobbezoo

**Affiliations:** 1 Department of Orofacial Pain and Dysfunction, Academic Centre for Dentistry Amsterdam (ACTA), University of Amsterdam and Vrije Universiteit Amsterdam, Gustav Mahlerlaan 3004, 1081 LA Amsterdam, The Netherlands; 2 Centre for Special Care in Dentistry (SBT), Gustav Mahlerlaan 3004, 1081 LA Amsterdam, The Netherlands; 3 Dagelijks Leven, Stationsstraat 122, 7311 EN Apeldoorn, The Netherlands; 4 Department of Clinical Neuropsychology, VU University Amsterdam, Van der Boechorststraat 1, 1081 BT Amsterdam, The Netherlands

**Keywords:** Intervention, Oral health care, Community dwelling, Dementia, Interdisciplinary collaborations, Health care professional

## Abstract

**Background:**

The oral health of community dwelling frail older people is poor, and depends on the oral health care provisions available within their own community. The implementation project ‘Don’t forget the mouth!’ (i.e.*,* the intervention) was created with the aim of maintaining and improving the oral health and the general health of community dwelling frail older people, with education and interdisciplinary collaborations of health care professionals and informal caregivers. Critical scientific assessment of the intervention will be described in this study protocol, focused on community dwelling older people with dementia.

**Methods:**

This protocol describes a prospective longitudinal single-blind multicentre study, which will take place in 14 towns, each with an intervention and a control group receiving oral health care as usual. Assessment will take place four times during 12 months (i.e.*, at baseline, after 3, 6, and 12 months*). Participants are 65 years and older, community dwelling, and suspected of dementia by their home care worker. The home care organizations will inform, and approach their clients about the study, before the researcher will reach out. The effectiveness of the intervention will be determined with the primary outcome variable ‘oral hygiene’, assessed through the presence of dental plaque (DP) or dental prosthetic plaque (DPP). The secondary outcome variables are: ‘oral health’, ‘oral health related quality of life’, ‘oral health care behaviour’, ‘general health and care dependency’, and ‘compliance of the health care professionals’.

**Discussion:**

This protocol aims to assess the effectiveness of the implementation project ‘Don’t forget the mouth!’ focused on community dwelling older people with dementia. The strengths of the current study are the national roll-out of the intervention, interdisciplinary collaborations and education, and the scientific evaluation over the course of 12 months. The threats and weaknesses are in the recruitment procedure, and the adherence and compliance of the health care professionals to the project.

**Trial registration:**

The Netherlands Trail Register NTR6159.

**Electronic supplementary material:**

The online version of this article (10.1186/s12903-019-0786-5) contains supplementary material, which is available to authorized users.

## Background

Older people are keeping their natural dentition [1] and are staying community dwelling, until high age [2]. The oral health of community dwelling frail older people is poor [3–5], increasing the risks of pneumonia [6], dysregulation of diabetes mellitus [7], and heart and cardiovascular diseases [8]. Additionally, poor oral health can impair mastication [9–11], which can be associated with malnutrition [10, 12], and cognitive decline [9, 10, 13].

Older people with dementia compared to people without dementia have poorer oral health, characterized by, amongst others, larger amounts of oral plaque and calculus, higher prevalence of mucosa deviations, presence of (root)caries, and an increased dental treatment need [5, 12, 14–16]. On top of that the frequency of visits to an oral health care professional decreases [14]. When older people become care dependent, e.g.*,* as with dementia, caregivers often do not have enough knowledge about how to provide oral hygiene care, nor do they have the proper skills to provide it [17, 18]. Inadequate prioritising and providing oral hygiene care by home care workers, case managers and physicians causes decline of oral health of people with dementia [3]. While the demand increases for (professional) oral health care in the community, the current oral health care system is not prepared to provide [11, 19, 20].

In 2014, a Dutch study [21] highlighted that although some promising national interventions were developed to improve oral health care of older people in nursing homes, only a few have focused on improving this aspect for community dwelling older people. In addition, the authors of the study strongly recommended scientifically evaluating all future interventions [21].

The implementation project ‘Don’t forget the mouth!’ was developed with the aim of maintaining and improving the oral health and the general health of community dwelling frail older people with education, and interdisciplinary collaborations of: physicians, general practice nurses, dentists, dental hygienists, case managers, district nurses, home care workers (i.e.*, health care professionals*), and informal caregivers.

Critical scientific assessment of this implementation project is described in this study protocol, focused on community dwelling older people with dementia.

## Methods and design

### Study design

This protocol describes a prospective longitudinal single-blind multicentre study, which will take place in 14 towns in the Netherlands. Recruitment will take place in two districts within a town, i.e.*, an intervention group* and *a control group*, that are matched based on social-economic status and population composition, and are enrolled through the town’s home care organization. Assignment of a district to the intervention or the control group will be done ‘a priori’. The intervention group will receive care of the healthcare professionals who participate in the implementation project ‘Don’t forget the mouth!’. The control group will receive care as usual. The participants will be visited at their home for assessment, at baseline, after 3, 6, and 12 months. The health care professionals of the intervention group will receive questionnaires. All data collection will be done by the same blinded researcher, viz., a dentist trained in the geriatric dentistry. Participants will be blinded. Health care professionals participate in the intervention, and therefore they cannot be blinded. The blinding code can be broken by the supporting investigator (RW) or principal investigator (FL) in case of a medical emergency of a participant. The Data Safety Monitoring Board (DSMB) is not needed because the risk for adverse side effects in this study is determined to be negligible. An independent monitor will monitor the study data according to ‘Good Clinical Practice’(GCP).

### Participants

The inclusion criteria are: community dwelling people aged 65 years or older; a Mini Mental State Examination (MMSE, a short screening instrument which assesses general cognition, awareness, and memory [22]) score between 18 and 24, which in this study will be considered indicative of dementia [23, 24], and receiving care from health care professionals of the participating home care organization and general practice. Exclusion criteria are: suffering from a terminal illness; having a MMSE score lower than 18 or higher than 25; and being edentulous without prosthesis. Additionally, the participants in the control group will be excluded if they are registered at the general or dental practice of the intervention group.

#### Recruitment

The home care organizations will inform clients with suspected dementia and their informal caregivers about the study, and will ask if the researcher may reach out to them. When permission is given, the client and their informal caregiver will be contacted by phone, after which an information letter will be sent. After a follow-up conversation by phone, a home visit will be planned to obtain written consent. At all times, participants will be able to withdraw or decline their participation.

### Health care professionals

In this study, the physicians, general practice nurses, dentists, dental hygienists, case managers, district nurses, and home care workers will be referred to as health care professionals. In order to be eligible to participate in this study, a health care professional, practice, or organization must also meet several inclusion and exclusion criteria. The inclusion criteria are: willing to include oral health care in the practice organization; the health care professional in the general health practice is willing to use validated frailty questionnaires [25–27]; the dentist has affinity with older people; the dental practice is accessible for older and/or disabled people [28]; and the health care professional of the home care organization is willing to make an inventory of the oral health care needs of their clients. The exclusion criteria are: temporary position of a health care professional; and providing only care to a population younger than 65 years.

#### Recruitment

All health care professionals are recruited by the project team of the implementation project ‘Don’t forget the mouth!’. Before the study starts, the health care professionals will be informed through an information letter, and written consent will be obtained. At all times, health care professionals will be able to withdraw or decline their participation in this study.

### Intervention

The implementation project ‘Don’t forget the mouth!’ *(*i.e.*, the intervention)* aims to maintain and improve the oral health and the general health of community dwelling frail older people, with education and interdisciplinary collaborations. The collaborations of the health care professionals will be focused on of early recognition of decrease of daily oral hygiene care, oral health problems, and referral to colleagues if needed. Over the course of 12 months, health care professionals will attend four different meetings: two regional, and two national. The regional meetings will focus on the regional organisation of oral health care by interdisciplinary collaboration, the attitude towards oral health care, and oral health. The national meetings will focus on education about providing daily oral hygiene care, oral hygiene care for non-cooperative patients or clients, oral health, and professional oral health care.

Educational materials about daily oral hygiene care, oral health and professional oral health care have been developed for the participating health care professionals, informal caregivers, and all community dwelling frail older people, and will be available online (www.demondnietvergeten.nl). The intervention will also provide a screening-referral tool, which will be used by all health care professionals.

## Outcome variables

### Primary outcome variables

#### Oral hygiene

The primary outcome variable, oral hygiene, will be determined by oral plaque assessments. The dental plaque (DP), which is the biofilm of oral plaque present on a participant’s natural dentition, fixed dental prosthetics, and/or dental implants, will be measured according to the Silness and Löe index [29]. The dental prosthesis plaque (DPP), which is the biofilm of oral plaque present on a participant’s removable dental prostheses (RDP), will be assessed according to the Augsburger and Elahi index [30]. Both oral plaque measuring methods will be applied simultaneously if a participant has a partial dentition and one or two RDP(s).

##### Dental plaque

The DP will be classified according to the Silness and Löe index (score range.

0–3, 0 = no plaque, 1 = plaque with probing, 2 = visible plaque without probing, and 3 = abundance of plaque more than one third of the buccal side) [29], on pre-specified teeth: two molars, two premolars, and two incisors, one in the maxilla and one in the mandible (i.e.*, the 16, 21, 24, 34, 41, and 46).* If one of the pre-specified teeth is missing, the tooth distal of the absent one will be assessed. If the distal element is also absent, the next present tooth mesial of the absent one in the same quadrant will be assessed. Deviations will be noted. The DP will be assessed using a dental mirror (mirror holder: Carl Martin, 485CH chroom, Solingen, Germany; mirror: Intertek, front rhodium nr. 3, New York, United States) and a dental probe (Intertek, CP12, New York, United States).

##### Dental prosthesis plaque

The DPP will be classified according to the Augsburger and Elahi index [30]. In this method, the maxillary RDP will be rinsed, coloured with a disclosing solution, a score will be given to each of the eight planes (score range 0–4, 0 = 0% plaque, 1 = 1–25% plaque, 2 = 26–50% plaque, 3 = 51–75% plaque, and 4 = 76–100% plaque, visible) [30], and a total score will be calculated. As disclosing solution, 0.05% methylene blue (produced by pharmacy ‘De Tolgaarde’, Leusden, Netherlands) will be used. In the current study, the assessment of the mandibular RDP has been added. The DPP score will be averaged in case there are two RDPs (an maxillary and a mandibular). To facilitate reproducibility, the RDP will be placed on a specially designed reference sheet with a grid outline indicating the planes. The RDP is then photographed with a dental camera (Shofu, Eye special II, Ratingen, Germany). A pilot study showed excellent inter-rater, and intra-rater agreement for both the maxillary and mandibular RDP assessments with the described method, when performed by trained and calibrated researchers (data not yet published).

### Secondary outcome variables

Secondary outcome variables are ‘oral health’, ‘oral health related quality of life’, ‘oral health care behaviour’, ‘general health and care dependency’, and ‘compliance of the health care professionals’.

#### Oral health

The oral health will be measured with the Oral Health Assessment Tool (OHAT) for dental screening [31], a validated screening tool for non-dental health professionals which assesses eight categories with regards to oral health: lips, tongue, gums and tissues, saliva, natural teeth, dentures, oral cleanliness, and dental pain. Each category is scored in a range from 0 to 2 (0 = a healthy situation, 1 = changes in the situation, and 2 = an unhealthy situation). All scores are summed; the final score is between 0 and 16, with a lower score indicating a healthier situation.

The OHAT by Chalmers (2005) was translated into Dutch using the forward-backward approach, in compliance with the ‘Guidelines for establishing cultural equivalency of instruments’ [32]. The translation from English to Dutch was done independently by two Dutch people who are fluent in both English and Dutch (BVH and RW; a dentist and a neurobiologist, respectively). Together with a third bilingual Dutch-native speaking person (FL, dentist and orofacial pain expert), consensus was reached. This version was back-translated into English by an external professional translator. The retranslated English version was compared to the original version, and the full team (BVH, RW, FL, and the professional translator) continued discussing discrepancies between the translations until consensus was reached on the final Dutch version (OHAT-NL, see Additional file 1: Appendix I).

#### Oral health related quality of life

The oral health related quality of life (OHRQoL) [33] will be measured with the Dutch version of the Geriatric Oral Health Assessment Index (GOHAI-NL) [34], a questionnaire which addresses three aspects of OHRQoL: physical function, psychosocial function, and pain/discomfort, with twelve items [34]. The simple count score (GOHAI-SC) [34, 35] will be used to dichotomize the answers for the participants. If needed, additional information from informal caregivers can be taken into account. The score options for the items are dichotomized as follows: 0 = never, seldom; 1 = sometimes, often, nearly always or always. The total score of the GOHAI-SC thus varies from 0 to 12. A higher score indicates a better experienced OHRQoL [34].

#### Oral health care behaviour

Information about the oral health care behaviour of the participants will be collected through two questionnaires designed for this study. The first questionnaire contains twelve items for the participant about daily oral hygiene care, difficulties with oral hygiene care, and visits to an oral health care professional. The second questionnaire contains fourteen items for the informal caregiver about their role, knowledge, and difficulties with oral health care of the participant.

#### General health and care dependency

General health and care dependency will be measured with The Older People and Informal Caregiver Survey Minimum Data Set (TOPICS-MDS) questionnaires [36], at baseline and after 12 months. These questionnaires are part of a larger national initiative to assess the Dutch senior population [36]. There are two questionnaires, one for the older person and one for the informal care giver. The questionnaire at baseline for the participant contains 51 items, the follow-up questionnaire contains 46 items; both are about demographics, morbidity, quality of life, functional limitations, emotional wellbeing, social functioning, and health service utilization [37]. The questionnaire at baseline for the informal care giver contains 27 items, the follow-up questionnaire contains 26 items; both are about demographics, hours of informal care, and quality of life [37].

Only the data collected with items about demographics, morbidity, health service utilization, and hours of informal care will be analysed in the current study to describe the population and compare the general health and care dependency between the intervention group and the control group.

#### Compliance of the health care professionals

To explore how the intervention is carried out, the compliance of the health care professionals will be assessed with questionnaires specifically designed for this study. There are six different questionnaires, three versions at baseline and three versions for the follow-up phase. There is one version for the health care professionals at the general practice, one version for the health care professionals at the dental practice, and one version for health care professionals of the home care organization. The questionnaires at baseline contain nine (home care organization) or ten (general practice and dental practice) items, about their care population, collaborations with other health care professionals, oral health care education, and oral health care organization in the practice or organization for frail older people.

The follow-up questionnaires contain eight (general practice) or nine (dental practice and home care organization) items, about the same subjects as mentioned at baseline; the item describing their care population will be left out.

### Sample size

The primary outcome variable is oral hygiene, determined by the proxy oral plaque, that will be classified according to the Silness and Loë index for dental plaque (DP) [29] and/or the Augsburger and Elahi index for dental prosthetic plaque (DPP) [30].

To calculate the sample size, a power of 80% was chosen (β = 0.20), with a significance level (α) of 0.05, expected standard deviation (σ) DP = 0.75 and DPP = 0.88 [3, 38]. For the intervention group, expected average scores were (μ^2^) DP = 1.63 and DPP = 1.60, for the control group the expected average scores were (μ^1^) DP = 2.17 and DPP = 2.13 [3, 38]. A clinically relevant effect of the intervention was set at an improvement of the oral hygiene with a DP and DPP reduction of 25% [3]. The sample size (*n*) was then calculated with the formula below [3].

$$ n=\frac{2\upsigma 2}{\left({\upmu}^1-{\upmu}^2\right)2}\ f\ \left(\alpha, \beta \right) $$

To take non-parametric testing, drop-outs and loss to follow up in account, the sample size will be increased with 10% [9]. As a result, a sample size of *n* = 34 for DP and *n* = 49 for DPP would suffice.

This study will have intervention groups and control groups, and will take place in multiple towns and therefore is considered a multicentre study. With that in mind the sample size formula should be adjusted to the design effect (i.e.*,* the different towns that are participating).

$$ Towns\ needed=\frac{2n\left(1+\left(m-1\right) ICC\right)}{m} $$

The expected intra class correlation is 0.03 [3, 38]. Based on a previous study [39], and the pilot study of ‘Don’t forget the mouth!’ (data not published), the expected number of participants (*m)* in each town is 20 in total, 8 for DP, and 12 for DPP. In total, at least 11 towns are needed in this study.

### Statistical analysis

The personal data will be handled according the Dutch Personal Data Protection Act [40]. The data of each participant will be coded. The codes will be safeguarded by the coordinating investigator (BVH) and the principal investigator (FL). Data will be safely stored in the data management programme Castor EDC [41], and will be kept for 15 years [40]. Only the principal investigator (FL) and coordinating investigator (BVH) can fully access the final trial dataset.

Baseline comparisons between the intervention group and control group will be made using the Mann-Whitney U test. Oral hygiene will be compared at baseline, after 3, 6, and 12 months follow-up for the effect of time using the Friedman test, and the effect of group over time (intervention versus control) with the Kruskal Wallis test. The oral health and OHRQoL will also be analysed with the Mann-Whitney U test and the Kruskal Wallis test. Oral health care and the compliance of the health care professionals will be analysed according the constant comparative method of qualitative analysis [42, 43]. General health care and care dependency, will be assessed with descriptive statistics. The analysis will be conducted with IBM SPSS Statistics version 25, the level of significance will be 0.05. Protocol non-adherence is not applicable in this design. In case of missing data, imputation techniques will not be applied.

## Discussion

This protocol describes a prospective longitudinal single-blind multicentre study, which aims to assess the effectiveness of the implementation project ‘Don’t forget the mouth!’ focused on community dwelling older people with dementia.

As any study, this protocol has some specific strengths, weaknesses, threats, and opportunities, which will be lined out below.

The strengths of the current study are the national roll-out of the intervention, interdisciplinary collaborations and education of different health care professionals focused on knowledge, attitude and skills of oral health care, and the scientific evaluation over the course of 12 months. According to the sample size calculation 11 towns are needed for this study, while the implementation project ‘Don’t forget the mouth!’ will take place in 14 towns, providing margin for withdraws.

The possible weaknesses of assessing the effectiveness of the implementation project are selection bias from allocation, due to recruitment through the home care organization. Randomization of the districts was not possible in the described study. Participants will not explicitly be made aware of their allocation, but some will be aware of the intervention as they are receiving educational materials through their health care professionals. Another possible weakness is the fact that the GOHAI-NL is not validated for people with dementia [34]. At the time of writing the protocol, there were no validated questionnaires available to assess OHRQoL for people with dementia. The reliability of the answers of the GOHAI-NL will be increased through taking, if needed, additional information from informal caregivers to account [34]. The two questionnaires about oral health care behaviour and compliance of the health care professionals are designed for this study, and not validated, which is a weakness of these instruments.

Within the current study, there is an opportunity to additionally assess the process of the implementation. If process evaluation can be conducted, recommendations about success factors or thresholds with implementation can be made to health care professionals.

The threats are found in the recruitment procedure (through the home care organisation), different informal caregivers with each assessment, and the adherence and compliance of the health care professionals to the intervention.

Despite the weaknesses and threats, this study protocol is unique, assessing the effectiveness of an oral health care implementation focused on community dwelling older people with dementia, while in current oral health care literature often this group is excluded. On top of that this is one of the few studies focused on interdisciplinary collaborations of health care professionals and informal caregivers for oral health care.

This study protocol gratifies the recommendation of scientifically evaluating all future interventions on improving oral health care for frail older people with or without dementia.

## Additional files

Additional file 1: OHAT-NL. (PDF 354 kb)

Additional file 2: Model information and consent form of the participants, informal caregivers, and health care professionals. (PDF 812 kb)

## Abbreviations

DP: Dental plaque
DPP: Dental prosthetic plaque
DSMB: Data Safety Monitoring Board
GCP: Good Clinical Practice
GOHAI: Geriatric Oral Health Assessment Index
GOHAI-SC: Geriatric Oral Health Assessment Index – simple count
MMSE: Mini Mental State Examination
OHAT: Oral Health Assessment Tool
OHAT-NL: Oral Health Assessment Tool – Dutch version
OHRQoL: Oral Health Related Quality of Life
RDP: Removable dental prosthesis
TOPICS-MDS: The Older Persons and Informal Caregiver Survey Minimum DataSet
