# Supplementary material for: Effectiveness of the implementation project ‘Don’t forget the mouth!’ of community dwelling older people with dementia: a prospective longitudinal single-blind multicentre study protocol (DFTM!)
Source: BMC Oral Health. 2019 May 28;19:91. doi: 10.1186/s12903-019-0786-5 (PMC6537198; doi:10.1186/s12903-019-0786-5)
Supplement: Supplementary file 1 — OHAT-NL. (PDF 354 kb) [file 12903_2019_786_MOESM1_ESM.pdf]

Appendix I

Oral Health Assessment Tool (OHAT) voor screening van de mond

gemodificeerd van Kayser-Jones et al (1995) door Chalmers (2005)

Bewoner:______________________________<br>Ingevuld door:______________________________ Functie/rol: ______________________________				Datum:__/___/__
**Categorie**	Scores – U kunt zowel individuele woorden omcirkelen als ook een score geven aan elke categorie<br>*(* als 1 of 2 wordt gescoord voor enige categorie: regel a.u.b. een tandarts die de bewoner onderzoekt)*			**Hoogste score per categorie**
	**0 = gezond**	**1 = veranderingen***	**2 = ongezond***	
Lippen	glad, roze, vochtig	droog, schraal of rode mondhoeken	zwelling of knobbel, witte/rode/zwerende plek; bloedende/zwerende mondhoeken	
Tong	normaal, vochtig, ruw, roze	gevlekt, gegroefd, rood, beslagen	rode en/of witte, zwerende of gezwollen plek	
Tandvlees en slijmvliezen	roze, vochtig, glad, geen bloeding	droog, glimmend, ruw, rood, gezwollen, één zweer/ pijnlijke plek onder kunstgebit	gezwollen, bloedend, zweren, witte/rode plekken, algemene roodheid onder kunstgebit	
Speeksel	vochtige weefsels, waterig en vrij-vloeiend speeksel	droog, plakkerige weefsels, weinig speeksel aanwezig, bewoner meent een droge mond te hebben	weefsels uitgedroogd en rood, zeer weinig/geen speeksel aanwezig, speeksel is dik, bewoner meent een droge mond te hebben	
Eigen tanden Ja/Nee	geen rotte of afgebroken tanden/wortels	1-3 rotte of afgebroken tanden/wortels of zeer afgesleten tanden	4+ rotte of afgebroken tanden/wortels, of zeer afgesleten tanden, of minder dan 4 tanden	
Kunstgebit Ja/Nee	geen kapotte gebieden of tanden, kunstgebit wordt regelmatig gedragen en is voorzien van naam	1 kapot gebied/tand, of kunstgebit wordt alleen gedragen gedurende 1-2 uur per dag, kunstgebit is niet voorzien van naam of zit los	meer dan 1 kapot gebied/tand, kunstgebit ontbreekt of wordt niet gedragen, zit los en heeft kleefpasta nodig of is niet voorzien van naam	
Mondhygiëne	schoon en geen voedseldeeltjes of tandsteen in de mond of op kunstgebit	voedseldeeltjes/tandsteen/ plaque in 1-2 gebieden van de mond of op klein gedeelte van kunstgebit aanwezig of halitosis (slechte adem)	voedseldeeltjes/tandsteen/ plaque in de meeste gebieden van de mond of kunstgebit of ernstige halitosis (slechte adem)	
Mondpijn	geen gedragsmatige, verbale of fysieke tekenen van mondpijn	er zijn verbale en/of gedragsmatige signalen van pijn zoals trekken aan het gezicht, kauwen op de lip, niet eten, agressie	er zijn zowel fysieke tekenen van pijn (zwelling van wang of tandvlees, gebroken tanden, zweren) als verbale en/of gedragsmatige signalen (trekken aan gezicht, niet eten, agressie)	
o Regel voor de bewoner een mondonderzoek door een tandarts<br>o Bewoner en/of familie/ wettelijk vertegenwoordiger weigert tandheelkundige behandeling<br>o Voltooi mondzorgplan en start mondzorginterventie voor bewoner<br>o Evalueer de mondgezondheid van deze bewoner opnieuw op Datum:____/____/____				TOTALE SCORE:

Nederlandse vertaling door ACTA afdeling Orale Kinesiologie
