# Supplementary material for: Effectiveness of the implementation project ‘Don’t forget the mouth!’ of community dwelling older people with dementia: a prospective longitudinal single-blind multicentre study protocol (DFTM!)
Source: BMC Oral Health. 2019 May 28;19:91. doi: 10.1186/s12903-019-0786-5 (PMC6537198; doi:10.1186/s12903-019-0786-5)
Supplement: Supplementary file 2 — Model information and consent form of the participants, informal caregivers, and health care professionals. (PDF 812 kb) [file 12903_2019_786_MOESM2_ESM.pdf]

Tot stand gekomen in het kader van het Deltaplan Dementie

Proefpersoneninformatie voor deelname onderzoek
Mondzorg voor ouderen

Geachte heer/mevrouw,

Wij vragen u vriendelijk om mee te doen aan een medisch-wetenschappelijk onderzoek.
U ontvangt deze brief omdat u toestemming heeft gegeven aan uw thuiszorgmedewerker dat wij contact met u mochten opnemen.

Aan dit onderzoek zullen minstens 176 proefpersonen in Nederland met kenmerken van dementie meedoen. Om het de proefpersonen makkelijk te maken, vindt het onderzoek plaats bij de mensen thuis.

Voordat u beslist of u wilt meedoen aan dit onderzoek, krijgt u uitleg over wat het onderzoek inhoudt. Lees deze informatie rustig door, bespreek het met uw partner, vrienden of familie. Als u na het lezen van de informatie nog vragen heeft kunt u terecht bij de onderzoekers.

Aanvullende informatie over meedoen aan een onderzoek staat in de bijgevoegde brochure 'Medisch-wetenschappelijk onderzoek', bijlage **E**.

1. Wat is het doel van het onderzoek?
Het doel van dit onderzoek is uitzoeken of extra training aan zorgverleners leidt tot verbetering van de mondgezondheid en algemene gezondheid bij thuiswonende ouderen met kenmerken van dementie.

2. Wat houdt dit onderzoek in?
Tijdens dit onderzoek krijgen zorgverleners extra training over de mondgezondheid en het verzorgen van de mond. Met zorgverleners worden huisarts, praktijkondersteuner huisarts, tandarts, casemanager dementie, wijkverpleegkundige en thuiszorgmedewerker bedoeld.

3. Hoe wordt het onderzoek uitgevoerd?
Er komen twee groepen. De helft van de proefpersonen krijgt zorg van de zorgverleners die de extra training hebben gevolgd. De andere helft krijgt de gebruikelijke zorg van de zorgverleners die deze training nog niet hebben gevolgd. U en de onderzoeker weten niet in welke groep u wordt geplaatst.

In één jaar tijd komen de onderzoekers vier keer bij u langs. Dat gebeurt op een dag en tijdstip dat u met ze heeft afgesproken. U en uw mantelzorger of familielid krijgen voor het 1e en 4e bezoek apart een algemene vragenlijst opgestuurd. We vragen u en uw mantelzorger of familielid deze van te voren in te vullen.
Het eerste bezoek duurt maximaal anderhalf uur, de drie bezoeken daarna duren maximaal een uur. Het is de bedoeling dat bij alle vier de bezoeken een mantelzorger of familielid erbij blijft.
Tijdens het 1e bezoek zal het volgende gebeuren: ondertekenen van de toestemmingsformulieren en het afnemen van een geheugentest. Daarna bekijkt de onderzoeker de eigen tanden en/of kunstgebit en vult u samen twee vragenlijsten over de mond in.

Bij de 3 volgende bezoeken zal dan het volgende gebeuren: de onderzoeker bekijkt de eigen tanden en/of kunstgebit en vult samen met u twee vragenlijsten over de mond in.

In bijlage **C** wordt uitgebreid uitgelegd wat er bij elk van de 4 bezoeken gebeurt.

4. Wat wordt er van u verwacht?
Om het onderzoek goed te laten verlopen is het belangrijk dat u zich aan de volgende afspraken houdt. De afspraken zijn dat u:

- niet ook nog aan een ander medisch-wetenschappelijk onderzoek meedoet.
- afspraken voor bezoeken nakomt.
- ondersteunt wordt door aanwezigheid van uw mantelzorger of familielid bij de bezoeken.

5. Wat is meer of anders dan de reguliere behandeling(en) die u krijgt?
Er zijn proefpersonen die extra mondzorg krijgen en er zijn proefpersonen die de reguliere mondzorg krijgen.

6. Welke bijwerkingen kunt u verwachten?
Uw kunstgebit wordt uit de mond genomen en besprenkeld met een veel gebruikt tijdelijke kleurstof voor kunstgebitten en daarna weer goed schoongemaakt. Er is een zeer kleine kans dat de tijdelijke kleurstof voor kunstgebitten een allergische reactie geeft. Mocht dit gebeuren kunt u contact opnemen met de onderzoeker, contactgegevens vindt u in bijlage **A**.

7. Wat zijn mogelijke voor- en nadelen van deelname aan dit onderzoek?
Voordelen van meedoen aan het onderzoek kunnen zijn

- een verbetering van uw mondgezondheid en algemene gezondheid door de extra training van de zorgverleners.

Nadelen van meedoen aan het onderzoek kunnen zijn

- het eerste bezoek duurt maximaal anderhalf uur;
- de drie andere bezoeken duren maximaal een uur;

een mogelijke bijwerking bij de metingen tijdens het onderzoek.

8. Wat gebeurt er als u niet wenst deel te nemen aan dit onderzoek?
U beslist zelf of u meedoet aan het onderzoek. U kunt zich altijd bedenken en toch stoppen, ook tijdens het onderzoek. U hoeft niet te zeggen waarom u stopt. De gegevens die tot dat moment zijn verzameld, worden gecodeerd gebruikt voor het onderzoek.
Het kan zijn dat uw mantelzorger of familielid opmerkt dat u zich tijdens het onderzoek verzet (niet meewerkt). De onderzoeker zal het onderzoek dan direct stoppen. Het is moeilijk om precies te omschrijven wat verzet is. Voor de start van het onderzoek wordt met u en uw mantelzorger overlegd wat als verzet wordt gezien.

9. Wat gebeurt er als het onderzoek is afgelopen?
Na het onderzoek worden de belangrijkste resultaten doorgegeven aan uw huisarts, tandarts en thuiszorg organisatie. De zorgverleners die nog niet de extra training hebben gekregen over de mondgezondheid en het verzorgen van de mond zullen deze nog krijgen.
Met de resultaten van dit onderzoek kan er in de toekomst wellicht een vervolgonderzoek worden uitgevoerd, u kunt op het toestemmingsformulier aangeven of u hiervoor benaderd mag worden.

10. Bent u verzekerd wanneer u aan het onderzoek meedoet?
Voor iedereen die meedoet aan dit onderzoek is een verzekering afgesloten. De verzekering dekt schade door deelname aan het onderzoek. Dit geldt voor schade die naar boven komt tijdens het onderzoek, of binnen vier jaar na het einde van het onderzoek. In bijlage **B** vindt u de verzekerde bedragen, de uitzonderingen en de adresgegevens van de verzekeraar.

11. Wordt u geïnformeerd als er tussentijds voor u relevante informatie over de studie bekend wordt?
Als er nieuwe informatie over het onderzoek bekend wordt die belangrijk voor u is, laat de onderzoeker dit aan u weten. U wordt dan gevraagd of u blijft meedoen.

12. Wat gebeurt er met uw gegevens?
Voor dit onderzoek is het nodig om uw medisch- en persoonsgegevens te verzamelen, dit wordt gecodeerd verwerkt. Uw naam en andere persoonsgegevens worden weggelaten.
Alle gegevens worden verwerkt en 15 jaar opgeslagen onder een onderzoeksnummer.
De gegevens kunnen gecodeerd voor nader onderzoek op gebied van mondzorg en ouderenzorg, binnen en buiten Europa worden gebruikt.
Daarnaast kan er om te controleren of het onderzoek goed en betrouwbaar uitgevoerd is een controleur die voor het Academisch Centrum Tandheelkunde Amsterdam (ACTA) werkt en de Inspectie voor de Gezondheidszorg uw medische- en persoonsgegevens inzien.
Algemene informatie hierover vindt u in de brochure ‘Medisch-wetenschappelijk onderzoek’, bijlage **E**.

13. Wordt uw huisarts en/of behandelend specialist geïnformeerd bij deelname?
Wij laten uw huisarts, tandarts en thuiszorgorganisatie schriftelijk weten dat u meedoet aan het onderzoek. Dit is voor uw eigen veiligheid. Als u dit niet goed vindt, kunt u niet meedoen aan dit onderzoek.

14. Welke informatie wordt er met uw zorgverleners gedeeld?
Om het onderzoek goed uit te kunnen voeren nemen we contact op met uw huisarts, tandarts en thuiszorgorganisatie om de volgende informatie op te vragen: ziektegeschiedenis, ziekte diagnoses, en medicatiegebruik. Deze gegevens worden gecodeerd verwerkt. Uw naam en andere persoonsgegevens worden weggelaten. Alleen het onderzoeksteam weet welke code u heeft.

15. Zijn er extra kosten of is er een vergoeding wanneer u besluit aan dit onderzoek mee te doen?
Nee, er zijn geen kosten en/of vergoedingen verbonden aan deelname van dit onderzoek.
Als er erge problemen in de mond worden gezien tijdens het onderzoek door de onderzoeker wordt u geadviseerd contact op te nemen met uw eigen (tand)arts. Mocht u geen eigen tandarts hebben dan kan de onderzoeker u vragen contact op te nemen met dr. Weijenberg. Dr. Weijenberg zal u een tandarts in uw eigen buurt adviseren. Daarnaast kan uw zorgverlener problemen in de mond tegenkomen en ook verwijzen naar de (tand)arts.
De kosten die gemaakt worden bij de (tand)arts, zouden ook zonder deelname gemaakt moeten worden. Dit kan wel tot een eigen bijdrage van de ziektekosten leiden.

16. Welke medisch-ethische toetsingscommissie heeft dit onderzoek goedgekeurd?
De Medisch Ethische Toetsingscommissie van het VU Medisch Centrum (METc VUmc) heeft dit onderzoek goedgekeurd. Meer informatie over de goedkeuring vindt u in de brochure 'Medisch-wetenschappelijk onderzoek' bijlage **E**.

17. Wilt u verder nog iets weten?
Bij vragen kunt terecht bij de onderzoekers, mw. Ho of dr. Weijenberg, de contactgegevens kunt u vinden in bijlage **A**.
Wilt u graag een onafhankelijk advies over meedoen aan dit onderzoek? Dan kunt u terecht bij een onafhankelijk deskundige. Zijn gegevens vindt u in bijlage **A**.
Bij klachten kunt u het beste terecht bij de klachtenfunctionaris van de VU en ACTA.
Alle contactgegevens vindt u in bijlage **A**.

18. Ondertekening informed consent
Na één week bedenktijd, wordt u gevraagd over deelname aan dit onderzoek. Indien u toestemming geeft, zullen wij u vragen de bijbehorende toestemmingsverklaring te tekenen bij het eerste bezoek. Met het tekenen geeft u aan dat u de informatie heeft begrepen en instemt met deelname aan het onderzoek.
De toestemmingsverklaring wordt door de tandarts-onderzoeker, mw. Ho, bewaard. U krijgt een kopie of een tweede exemplaar van deze toestemmingsverklaring.

Met vriendelijke groeten,

Mw. Bach Van Ho, tandarts-onderzoeker
dr. Roxane Weijenberg, onderzoeker
prof. dr. Frank Lobbezoo, supervisor

Bijlagen: A contactgegevens, **B** informatie over de verzekering, **C** overzicht bezoeken en metingen, **D** toestemmingsformulier onderzoek proefpersoon en **E** brochure 'Medisch-wetenschappelijk onderzoek.'

Bijlage A: contactgegevens

Onderzoeker:	Mw. Bach Van Ho, tandarts-onderzoeker
Correspondentie adres:	[redacted]
E-mail:	[redacted]
Telefoonnummer:	[redacted]
Telefonisch bereikbaar:	[redacted]

2de aanspreekpunt:	dr. Roxane Weijenberg, onderzoeker
Correspondentie adres:	[redacted]
E-mail:	[redacted]
Telefoonnummer:	[redacted]
Telefonisch bereikbaar:	[redacted]

Onafhankelijk deskundige:	dr. Peter Wetselaar, tandarts-onderzoeker
Correspondentie adres:	[redacted]
E-mail:	[redacted]
Telefoonnummer:	[redacted]
Telefonisch bereikbaar:	[redacted]

Bij afwezigheid kunt u contact op nemen met het Secretariaat van afdeling Orale Kinesiologie

Secretaresse:	[redacted]
E-mail:	[redacted]
Telefoonnummer:	[redacted]
Telefonisch bereikbaar:	[redacted]

Klachten:	Klachtenfunctionaris van de VU en ACTA
Correspondentieadres:	[redacted]
Telefoonnummer:	[redacted]
Telefonisch beschikbaar:	[redacted]
Website met klachtenformulier:	[redacted]

Bijlage B: informatie over de verzekering

Voor iedereen die meedoet aan dit onderzoek, heeft het Academisch Centrum Tandheelkunde Amsterdam (ACTA) een verzekering afgesloten. De verzekering dekt schade door deelname aan het onderzoek. Dit geldt voor schade tijdens het onderzoek of binnen vier jaar na het einde ervan. Schade moet u binnen die vier jaar aan de verzekeraar hebben gemeld.

De verzekering dekt niet alle schade. Onderaan deze tekst staat in het kort welke schade niet wordt gedekt.
Deze bepalingen staan in het Besluit verplichte verzekering bij medisch-wetenschappelijk onderzoek met mensen. Dit besluit staat op www.ccmo.nl, de website van de Centrale Commissie Mensgebonden Onderzoek (zie ‘Bibliotheek’ en dan ‘Wet- en regelgeving’).

Bij schade kunt u direct contact leggen met de verzekeraar.
Dit is mogelijk via de website van de verzekeringsmaatschappij [redacted], u kunt natuurlijk ook een brief sturen naar onderstaande adres. In beide gevallen dient u het onderstaande polisnummer van de ACTA te vermelden.

De verzekeraar van het onderzoek is:	
Naam:	[redacted]
Adres:	[redacted]
Telefoonnummer:	[redacted]
Polisnummer:	[redacted]

De verzekering biedt een dekking van € 650.000 per proefpersoon maximaal en €5.000.000 voor het hele onderzoek en € 7.500.000 per jaar voor alle onderzoeken van dezelfde opdrachtgever.

De verzekering dekt de volgende schade **niet**:
- schade door een risico waarover u in de schriftelijke informatie bent ingelicht. Dit geldt niet als het risico zich ernstiger voordoet dan was voorzien of als het risico heel onwaarschijnlijk was;
- schade aan uw gezondheid die ook zou zijn ontstaan als u niet aan het onderzoek had meegedaan;
- schade door het niet (volledig) opvolgen van aanwijzingen of instructies;
- schade aan uw nakomelingen, als gevolg van een negatief effect van het onderzoek op u of uw nakomelingen;
- schade door een bestaande behandelmethode bij onderzoek naar bestaande behandelmethoden.

Bijlage C: overzicht bezoeken en metingen

Tabel 1 Overzicht van de bezoeken

Bezoek	Wat gaan we doen?	Maximale duur	Uitvoerder
	Algemene vragenlijst	30 minuten	U en uw mantelzorger of familielid
**1e bezoek**	- Toestemmingsformulieren ondertekenen<br>- Bespreken wat we tijdens het bezoek gaan doen<br>- Geheugentest*<br>- Tanden en/of kunstgebit bekijken**<br>- Algemene vragenlijst doornemen<br>- Vragenlijst over de mond<br>- Vragenlijst over de mondverzorging	10 minuten<br>10 minuten<br>30 minuten<br>10 minuten<br>10 minuten<br>10 minuten<br>10 minuten	Twee onderzoekers
	totaal	90 minuten	
**2e bezoek**	- Bespreken wat we tijdens het bezoek gaan doen<br>- Tanden en/of kunstgebit bekijken**<br>- Vragenlijst over de mond<br>- Vragenlijst over de mondverzorging	10 minuten<br>10 minuten<br>10 minuten<br>10 minuten	Twee onderzoekers
	totaal	40 minuten	
**3e bezoek**	- Bespreken wat we tijdens het bezoek gaan doen<br>- Tanden en/of kunstgebit bekijken**<br>- Vragenlijst over de mond<br>- Vragenlijst over de mondverzorging	10 minuten<br>10 minuten<br>10 minuten<br>10 minuten	Twee onderzoekers
	Totaal	40 minuten	
	Algemene vragenlijst	30 minuten	U en uw mantelzorger of familielid
**4e bezoek**	- Bespreken wat we tijdens het bezoek gaan doen<br>- Tanden en/of kunstgebit bekijken**<br>- Algemene vragenlijst doornemen<br>- Vragenlijst over de mond<br>- Vragenlijst over de mondverzorging	10 minuten<br>10 minuten<br>10 minuten<br>10 minuten<br>10 minuten	Twee onderzoekers
	totaal	50 minuten	

*/** zie volgende bladzijde

*** Geheugentest**
Met de geheugentest wordt er onder anderen gekeken naar het geheugen en de concentratie.

**** Tanden en/of kunstgebit bekijken**
Tanden en/of kunstgebit bekijken houdt in dat de onderzoeker in uw mond gaat kijken.
De onderzoeker bekijkt de tanden met een mondspiegeltje en een ‘tandarts haakje’.
Het kunstgebit wordt uit de mond genomen en dan besprenkeld met een tijdelijke kleurstof voor kunstgebitten. Het kunstgebit wordt op de foto gezet. Daarna wordt het kunstgebit goed schoongemaakt.

Bijlage D: toestemmingsformulier onderzoek proefpersoon

Toestemmingsformulier Onderzoek Mondzorg voor ouderen
Voor de proefpersoon

Ik heb de informatiebrief voor de proefpersoon gelezen. Ik kon aanvullende vragen stellen. Mijn vragen zijn genoeg beantwoord. Ik had genoeg tijd om te beslissen of ik meedoe.

Ik weet dat meedoen helemaal vrijwillig is. Ik weet dat ik op ieder moment kan beslissen om toch niet mee te doen. Daarvoor hoef ik geen reden te geven.

Ik geef toestemming voor het informeren van mijn huisarts, tandarts en thuiszorgorganisatie dat ik meedoe aan dit onderzoek.

Ik geef toestemming voor het opvragen van informatie bij mijn mantelzorger en/of familielid, huisarts, tandarts, en thuiszorgorganisatie over: mijn ziekte geschiedenis, ziekte diagnoses, en medicatiegebruik.

Ik weet dat sommige mensen mijn gegevens kunnen zien. Die mensen staan vermeld in de informatiebrief.

Ik geef toestemming om mijn gegevens te gebruiken, voor de doelen die in de informatiebrief staan.

Ik geef toestemming om mijn onderzoeksgegevens 15 jaar na afloop van dit onderzoek te bewaren.

Ik geef toestemming om mijn gegevens gecodeerd over te dragen naar landen buiten de Europese Unie waar de Europese richtlijnen voor de bescherming van persoonsgegevens niet gelden. Dit gebeurt alleen bij noodzakelijkheid voor het onderzoek en nader onderzoek op gebied van mondzorg en ouderenzorg. De gegevens moeten gecodeerd worden overgedragen en zonder mijn naam.

Ik geef ◻ **wel**
◻ **geen** toestemming om mij na dit onderzoek opnieuw te benaderen voor een vervolgonderzoek.

Ik wil ◻ **wel**
◻ **niet** geïnformeerd worden over in welke groep ik zat.

Ik wil meedoen aan dit onderzoek.

Naam proefpersoon:
Handtekening: Datum : __ / __ / __

(Dit gedeelte zal de onderzoeker ondertekenen)

Ik verklaar hierbij dat ik deze proefpersoon volledig heb geïnformeerd over het genoemde onderzoek.

Als er tijdens het onderzoek informatie bekend wordt die de toestemming van de proefpersoon zou kunnen beïnvloeden, dan breng ik hem/haar daarvan tijdig op de hoogte.

Naam onderzoeker (of diens vertegenwoordiger):
Handtekening: Datum: __ / __ / __

Aanvullende informatie is gegeven door (indien van toepassing):
Naam:
Functie:
Handtekening: Datum: __ / __ / __

Informatie voor deelname mantelzorger en/of familielid

Mondzorg voor ouderen

Geachte heer/mevrouw,

U bent mantelzorger en/of familielid van een oudere met mogelijke kenmerken van dementie. Wij vragen de persoon waar u voor zorgt vriendelijk om mee te doen aan een medisch-wetenschappelijk onderzoek. Graag zouden wij u willen vragen om aanwezig te zijn bij de bezoeken. Ook vragen we uw toestemming om een aantal vragenlijsten in te vullen met de persoon waar u voor zorgt en de onderzoekers.

We willen u graag meer informatie geven en vragen ook om uw toestemming voor uw eigen deelname aan dit onderzoek.

Lees deze informatie rustig door en bespreek het met uw partner, vrienden of familie. Als u na het lezen van de informatie nog vragen heeft, dan kunt u terecht bij de onderzoekers.

Aanvullende informatie over meedoen aan een onderzoek staat in de bijgevoegde brochure ‘Medisch-wetenschappelijk onderzoek’, bijlage **E**.

1. Wat is het doel van het onderzoek?

Het doel van dit onderzoek is uitzoeken of extra training aan zorgverleners leidt tot verbetering van de mondgezondheid en algemene gezondheid bij thuiswonende ouderen met kenmerken van dementie.

2. Wat wordt er van u verwacht?

In één jaar tijd komen twee onderzoekers vier keer bij de persoon waar u voor zorgt thuis langs. Dat gebeurt op een dag en tijdstip die u met ze heeft afgesproken. De persoon waar u voor zorgt en uzelf krijgen voor het 1e en 4e bezoek apart een algemene vragenlijst opgestuurd. We vragen de persoon waar u voor zorgt en u de vragenlijsten van te voren in te vullen. In de vragenlijst voor u worden er ook vragen gesteld over uw rol als mantelverzorger en/of familielid en uw ervaring met deze rol.

Het eerste bezoek duurt maximaal anderhalf uur; de drie bezoeken daarna duren maximaal een uur. Het is de bedoeling dat u ook bij alle vier de bezoeken aanwezig bent.
Tijdens het bezoek bekijkt de onderzoeker de tanden en/of kunstgebit van de persoon waar u voor zorgt en worden een aantal vragenlijsten over de mond ingevuld. Tijdens het bezoek wordt er ook aan u vragen gesteld over de mondverzorging van de persoon waar u voor zorgt en uw (eventuele) rol hierin.

In bijlage **C** wordt uitgebreid uitgelegd wat er bij elk van de 4 bezoeken gebeurt.

3. Wat gebeurt er als u niet wenst deel te nemen aan dit onderzoek?
U beslist zelf of u meedoet aan het onderzoek. U kan zich altijd bedenken en toch stoppen, ook tijdens het onderzoek. U hoeft niet te zeggen waarom u stopt. De gegevens die tot dat moment zijn verzameld, worden gecodeerd gebruikt voor het onderzoek.

Als de persoon waar u voor zorgt niet door u of een ander mantelzorger/familielid ondersteund kan worden bij het onderzoek, is het niet mogelijk voor hem/haar deel te nemen aan het onderzoek.

4. Wat gebeurt er als het onderzoek is afgelopen?
Met de resultaten van dit onderzoek kan er in de toekomst wellicht een vervolgonderzoek worden uitgevoerd. U kunt op het toestemmingsformulier aangeven of u hiervoor benaderd mag worden.

5. Bent u verzekerd bij deelname aan het onderzoek?
Voor iedereen die meedoet aan dit onderzoek is een verzekering afgesloten. De verzekering dekt schade door deelname aan het onderzoek. Dit geldt voor schade die naar boven komt tijdens het onderzoek, of binnen vier jaar na het einde van het onderzoek. In bijlage **B** vindt u de verzekerde bedragen, de uitzonderingen en de adresgegevens van de verzekeraar.

6. Wordt u geïnformeerd als er tussentijds relevante informatie over de studie bekend wordt?
Als er nieuwe informatie over het onderzoek bekend wordt die belangrijk is, laat de onderzoeker dit aan u weten. U wordt dan gevraagd of u en de persoon waar u voor zorgt blijven meedoen.

7. Wat gebeurt er met uw gegevens?
Alle gegevens worden verwerkt en 15 jaar opgeslagen onder een onderzoeksnummer. Alleen de onderzoekers van het onderzoeksteam weten welke code u heeft. De gegevens kunnen gecodeerd voor nader onderzoek op gebied van mondzorg en ouderenzorg worden gebruikt, zowel binnen als buiten Europa.
Daarnaast kan er om te controleren of het onderzoek goed en betrouwbaar uitgevoerd is een controleur die voor het Academisch Centrum Tandheelkunde Amsterdam (ACTA) werkt en de Inspectie voor de Gezondheidszorg uw persoonsgegevens inzien. Algemene informatie hierover vindt u in de brochure ‘Medisch-wetenschappelijk onderzoek’, bijlage **E**.

8. Zijn er extra kosten/is er een vergoeding wanneer u besluit aan dit onderzoek mee te doen?
Nee, er zijn geen kosten en/of vergoedingen verbonden aan deelname van dit onderzoek.

9. Welke medisch-ethische toetsingscommissie heeft dit onderzoek goedgekeurd?
Toetsingscommissie van het VU Medisch Centrum (METc VUmc) heeft dit onderzoek goedgekeurd. Meer informatie over de goedkeuring vindt u in de brochure ‘Medisch-wetenschappelijk onderzoek’, bijlage **E**.

10. Wilt u verder nog iets weten?
Bij vragen kunt u terecht bij de onderzoekers, mw. Ho of dr. Weijenberg, contactgegevens kunt u vinden in bijlage **A**.
Wilt u graag een onafhankelijk advies over meedoen aan dit onderzoek? Dan kunt u terecht bij een onafhankelijk deskundige. Zijn gegevens vindt u in bijlage **A**.
Bij klachten kunt u het beste terecht bij de klachtenfunctionaris van de VU en ACTA.
Alle contactgegevens vindt u in bijlage **A**.

11. Ondertekening informed consent
Na één week bedenktijd, wordt uw mening gevraagd over deelname aan dit onderzoek. Indien u toestemming geeft, zullen wij u vragen de bijbehorende toestemmingsverklaring te tekenen bij het eerste bezoek. Met het tekenen geeft u aan dat u de informatie heeft begrepen, en instemt met deelname als mantelzorger en/of familielid.
De toestemmingsverklaring wordt door de tandarts-onderzoeker, mw. Ho, bewaard. U krijgt een kopie of een tweede exemplaar van deze toestemmingsverklaring.

Met vriendelijke groeten,

Mw. Bach Van Ho, tandarts-onderzoeker
dr. Roxane Weijenberg, onderzoeker
prof. dr. Frank Lobbezoo, supervisor

Bijlagen: **A** contactgegevens, **B** informatie over de verzekering, **C** overzicht bezoeken en metingen, **D** toestemmingsformulier onderzoek mantelverzorger en/of familielid, en **E** brochure ‘Medisch wetenschappelijk onderzoek.’

Bijlage A: contactgegevens

Onderzoeker:	Mw. Bach Van Ho, tandarts-onderzoeker
Correspondentie adres:	[redacted]
E-mail:	[redacted]
Telefoonnummer:	[redacted]
Telefonisch bereikbaar:	[redacted]

2de aanspreekpunt:	dr. Roxane Weijenberg, onderzoeker
Correspondentie adres:	[redacted]
E-mail:	[redacted]
Telefoonnummer:	[redacted]
Telefonisch bereikbaar:	[redacted]

Onafhankelijk deskundige:	dr. Peter Wetselaar, tandarts-onderzoeker
Correspondentie adres:	[redacted]
E-mail:	[redacted]
Telefoonnummer:	[redacted]
Telefonisch bereikbaar:	[redacted]

Bij afwezigheid kunt u contact op nemen met het Secretariaat van afdeling Orale Kinesiologie	
Secretaresse:	[redacted]
E-mail:	[redacted]
Telefoonnummer:	[redacted]
Telefonisch bereikbaar:	[redacted]

Klachten:	Klachtenfunctionaris van de VU en ACTA
Correspondentieadres:	[redacted]
Telefoonnummer:	[redacted]
Telefonisch beschikbaar:	[redacted]
Website met klachtenformulier:	[redacted]

Bijlage B: informatie over de verzekering

Voor iedereen die meedoet aan dit onderzoek, heeft het Academisch Centrum Tandheelkunde Amsterdam (ACTA) een verzekering afgesloten. De verzekering dekt schade door deelname aan het onderzoek. Dit geldt voor schade tijdens het onderzoek of binnen vier jaar na het einde ervan. Schade moet u binnen die vier jaar aan de verzekeraar hebben gemeld.

De verzekering dekt niet alle schade. Onderaan deze tekst staat in het kort welke schade niet wordt gedekt.
Deze bepalingen staan in het Besluit verplichte verzekering bij medisch-wetenschappelijk onderzoek met mensen. Dit besluit staat op www.ccmo.nl, de website van de Centrale Commissie Mensgebonden Onderzoek (zie 'Bibliotheek' en dan 'Wet- en regelgeving').

Bij schade kunt u direct contact leggen met de verzekeraar.
Dit is mogelijk via de website van de verzekeringsmaatschappij [redacted], u kunt natuurlijk ook een brief sturen naar onderstaande adres. In beide gevallen dient u het onderstaande polisnummer van de ACTA te vermelden.

De verzekeraar van het onderzoek is:	
Naam:	[redacted]
Adres:	[redacted]
Telefoonnummer:	[redacted]
Polisnummer:	[redacted]

De verzekering biedt een dekking van € 650.000 per proefpersoon maximaal en €5.000.000 voor het hele onderzoek en € 7.500.000 per jaar voor alle onderzoeken van dezelfde opdrachtgever.

De verzekering dekt de volgende schade **niet**:

- schade door een risico waarover u in de schriftelijke informatie bent ingelicht. Dit geldt niet als het risico zich ernstiger voordoet dan was voorzien of als het risico heel onwaarschijnlijk was;
- schade aan uw gezondheid die ook zou zijn ontstaan als u niet aan het onderzoek had meegedaan;
- schade door het niet (volledig) opvolgen van aanwijzingen of instructies;
- schade aan uw nakomelingen, als gevolg van een negatief effect van het onderzoek op u of uw nakomelingen;
- schade door een bestaande behandelmethode bij onderzoek naar bestaande behandelmethoden.

Bijlage C: overzicht bezoeken en metingen

Tabel 1 Overzicht van de bezoeken

Bezoek	Wat gaan we doen bij de persoon waar u voor zorgt?	Maximale duur	Uitvoerder
	Algemene vragenlijst	30 minuten	De persoon waar u voor zorgt en u
1e bezoek	- Toestemmingsformulieren ondertekenen<br>- Bespreken wat we tijdens het bezoek gaan doen<br>- Geheugentest*<br>- Tanden en/of kunstgebit bekijken**<br>- Algemene vragenlijst doornemen<br>- Vragenlijst over de mond<br>- Vragenlijst over de mondverzorging	10 minuten<br>10 minuten<br>30 minuten<br>10 minuten<br>10 minuten<br>10 minuten<br>10 minuten	Twee onderzoekers
	totaal	90 minuten	
2e bezoek	- Bespreken wat we tijdens het bezoek gaan doen<br>- Tanden en/of kunstgebit bekijken**<br>- Vragenlijst over de mond<br>- Vragenlijst over de mondverzorging	10 minuten<br>10 minuten<br>10 minuten<br>10 minuten	Twee onderzoekers
	totaal	40 minuten	
3e bezoek	- Bespreken wat we tijdens het bezoek gaan doen<br>- Tanden en/of kunstgebit bekijken**<br>- Vragenlijst over de mond<br>- Vragenlijst over de mondverzorging	10 minuten<br>10 minuten<br>10 minuten<br>10 minuten	Twee onderzoekers
	Totaal	40 minuten	
	Algemene vragenlijst	30 minuten	De persoon waar u voor zorgt en u
4e bezoek	- Bespreken wat we tijdens het bezoek gaan doen<br>- Tanden en/of kunstgebit bekijken**<br>- Algemene vragenlijst doornemen<br>- Vragenlijst over de mond<br>- Vragenlijst over de mondverzorging	10 minuten<br>10 minuten<br>10 minuten<br>10 minuten<br>10 minuten	Twee onderzoekers
	totaal	50 minuten	

*/** zie volgende bladzijde

*** Geheugentest**
Met de geheugentest wordt er onder anderen gekeken naar het geheugen en de concentratie.

**** Tanden en/of kunstgebit bekijken**
Tanden en/of kunstgebit bekijken houdt in dat de onderzoeker in de mond van de persoon waar u voor zorgt gaat kijken.
De onderzoeker bekijkt de tanden met een mondspiegeltje en een ‘tandarts haakje’.
Het kunstgebit wordt uit de mond genomen en dan besprenkeld met een tijdelijke kleurstof voor kunstgebitten. Het kunstgebit wordt op de foto gezet. Daarna wordt het kunstgebit goed schoongemaakt.

Bijlage D: toestemmingsformulier onderzoek mantelverzorger en/of familielid

Toestemmingsformulier Onderzoek Mondzorg voor ouderen
Voor de mantelverzorger en/of familielid

Naam proefpersoon: Geboortedatum: __ / __ / __

Ik heb de informatiebrief voor de mantelverzorger en/of familielid gelezen. Ik kon aanvullende vragen stellen. Deze vragen zijn naar tevredenheid beantwoord. Ik heb voldoende tijd gehad om te beslissen of ik meedoe.

Ik weet dat meedoen helemaal vrijwillig is. Ik weet dat ik op ieder moment kan beslissen om toch niet mee te doen. Daarvoor hoef ik geen reden te geven.

Ik weet dat sommige mensen mijn gegevens kunnen zien. Die mensen staan vermeld in de informatiebrief.

Ik geef toestemming om mijn gegevens te gebruiken, voor de doelen die in de informatiebrief staan.

Ik geef toestemming om mijn onderzoeksgegevens 15 jaar na afloop van dit onderzoek te bewaren.

Ik geef toestemming om mijn gegevens gecodeerd over te dragen naar landen buiten de Europese Unie waar de Europese richtlijnen voor de bescherming van persoonsgegevens niet gelden. Dit gebeurt alleen bij noodzakelijkheid voor het onderzoek en nader onderzoek op gebied van mondzorg en ouderenzorg. De gegevens moeten gecodeerd worden overgedragen en zonder mijn naam.

Ik geef □ **wel**
□ **geen** toestemming om mij na dit onderzoek opnieuw te benaderen voor een vervolgonderzoek.

Ik wil □ **wel**
□ **niet** geïnformeerd worden over in welke groep ik zat.

Ik wil meedoen aan dit onderzoek.

Naam mantelverzorger en/of familie:
Handtekening: Datum : __ / __ / __

(Dit gedeelte zal de onderzoeker ondertekenen)

Ik verklaar hierbij dat ik deze proefpersoon volledig heb geïnformeerd over het genoemde onderzoek.

Als er tijdens het onderzoek informatie bekend wordt die de toestemming van de proefpersoon zou kunnen beïnvloeden, dan breng ik hem/haar daarvan tijdig op de hoogte.

Naam onderzoeker (of diens vertegenwoordiger):
Handtekening: Datum: __ / __ / __

Aanvullende informatie is gegeven door (indien van toepassing):
Naam:
Functie:
Handtekening: Datum: __ / __ / __

Tot stand gekomen in het kader van het Deltaplan Dementie

Zorgverlenersinformatie voor deelname onderzoek
Het implementatieproject 'De mond niet vergeten!'

Geachte heer/mevrouw,

U ontvangt deze brief omdat u beroepsmatig geïnteresseerd bent in deelname aan het implementatieproject 'De mond niet vergeten!' en het hieraan gekoppelde medisch-wetenschappelijk onderzoek.

Aan dit onderzoek zullen naar verwachting minstens 176 proefpersonen in Nederland met kenmerken van dementie meedoen. Om het de proefpersonen makkelijk te maken, vindt het onderzoek plaats bij de mensen thuis.

Met deze brief vragen we om uw deelname. U krijgt in deze brief meer informatie over het onderzoek. Lees deze informatie rustig door en, bespreek het met uw partner, vrienden of familie. Als u na het lezen van de informatie nog vragen heeft, kunt u terecht bij de onderzoekers.

Aanvullende informatie over meedoen aan een onderzoek staat in de bijgevoegde brochure 'Medisch-wetenschappelijk onderzoek', bijlage D.

1. Wat is het doel van het onderzoek?
Het doel van dit onderzoek is uitzoeken of het project 'De mond niet vergeten!' leidt tot verbetering van de mondgezondheid en algemene gezondheid bij thuiswonende ouderen met kenmerken van dementie.

2. Wat houdt het implementatieproject 'De mond niet vergeten!' in?
Tijdens dit project krijgen zorgverleners verbeterde scholing gefocust op het behoud en verbeteren van de mondgezondheid en de algemene gezondheid van thuiswonende ouderen met kenmerken van dementie. Daarnaast wordt de multidisciplinaire samenwerking ondersteund en bevorderd. Met zorgverleners worden huisarts, praktijkondersteuner huisarts, tandarts, casemanager dementie, wijkverpleegkundige en thuiszorgmedewerker bedoeld.

3. Hoe wordt het onderzoek uitgevoerd?
Proefpersonen worden in twee groepen verdeeld. De helft van de proefpersonen krijgt zorg van de zorgverleners die deelnemen aan het implementatieproject 'De mond niet vergeten!'. De andere helft krijgt de gebruikelijke zorg van de zorgverleners die deze training (nog) niet hebben gevolgd.
De proefpersonen weten <u>niet</u> of hun eigen zorgverleners deelnemen aan het implementatie-project 'De mond niet vergeten!'.

In één jaar tijd zullen de onderzoekers vier keer bij de proefpersoon op bezoek gaan op een tijdstip dat van te voren is afgesproken met de proefpersoon en zijn/haar mantelzorger en/of familielid. Tijdens deze bezoeken worden er meerdere metingen gedaan en vragenlijsten afgenomen in aanwezigheid van een mantelzorger of familielid.
Bij het vaststellen van urgente problemen in de mond zal de onderzoeker de proefpersoon en diens mantelzorger en/of familielid adviseren naar de (tand)arts te gaan.

4. Wat wordt er van u verwacht?

U neemt als zorgverlener deel aan het implementatieproject ‘De mond niet vergeten!’.
Om het onderzoek goed te doen verlopen zouden wij u willen vragen om op vier momenten in het jaar een vragenlijst met 8-10 vragen over de mondzorg in uw werkomgeving en de samenwerkingsverbanden in te vullen. Het invullen van deze vragenlijsten zal zoveel mogelijk gecombineerd worden met de bijeenkomsten van het van het implementatieproject ‘De mond niet vergeten!’.

Het is belangrijk dat het onderzoek ethisch en zo weinig mogelijk belastend voor de proefpersonen wordt uitgevoerd. Deze uitvoering wordt getoetst door de Medisch Ethische Toetsingscommissie van het VU Medisch Centrum (METc VUmc). De commissie en het onderzoeksteam zouden u willen vragen ten behoeve van deze kwaliteitszorg het volgende document te ondertekenen:

- Toestemmingsformulier; voor het verzamelen en anoniem verwerken van uw antwoorden op de vragenlijsten over de mondzorg in uw werkomgeving en samenwerkingsverbanden, bijlage **C**.

5. Wat zijn mogelijke voor- en nadelen van deelname aan dit onderzoek?

Voordelen van meedoen aan het onderzoek kunnen zijn:

- u krijgt scholing gefocust op de mondgezondheid en algemene gezondheid bij thuiswonende ouderen met dementie.
- (versterkte) samenwerkingsverbanden met zorgverleners uit de omgeving.
- dat u samen met het implementatieproject ‘De mond niet vergeten!’ een verbetering geeft aan de mondgezondheid en algemene gezondheid van uw patiëntenpopulatie.

Nadelen van meedoen aan het onderzoek kunnen zijn:

- het bijwonen van de scholing vraagt van u een tijdsinvestering.
- het invullen van de vragenlijst duurt ongeveer 15 minuten per keer (8-10 vragen).

6. Wat gebeurt er als u niet wenst deel te nemen aan dit onderzoek?

U beslist zelf of u meedoet aan het implementatieproject ‘De mond niet vergeten!’ en het bijbehorende onderzoek. U kunt zich altijd bedenken en toch stoppen, ook tijdens het onderzoek. U hoeft niet te zeggen waarom u stopt. De gegevens die tot dat moment zijn verzameld, worden gecodeerd gebruikt voor het onderzoek. Let hierbij wel op: deelname aan het onderzoek is een voorwaarde voor deelname aan het implementatieproject.

7. Wat gebeurt er als het onderzoek is afgelopen?

Na afloop van het onderzoek worden de belangrijkste resultaten aan u doorgegeven. De zorgverleners die nog niet de extra training hebben gekregen over de mondgezondheid en het verzorgen van de mond zullen deze als nog krijgen.
Met de resultaten van dit onderzoek kan er in de toekomst wellicht een vervolgonderzoek worden uitgevoerd. U kunt op het toestemmingsformulier aangeven of u hiervoor benaderd mag worden.

8. Bent u verzekerd wanneer u aan het onderzoek meedoet?

Voor iedereen die meedoet aan dit onderzoek is een verzekering afgesloten. De verzekering dekt schade door deelname aan het onderzoek. Dit geldt voor schade die naar boven komt tijdens het onderzoek, of binnen vier jaar na het einde van het onderzoek. In bijlage **B** vindt u de verzekerde bedragen, de uitzonderingen en de adresgegevens van de verzekeraar.

9. Wordt u geïnformeerd als er tussentijds voor u relevante informatie over de studie bekend wordt?
Als er nieuwe informatie over het onderzoek bekend wordt voor u is, laat de onderzoeker dit aan u weten. U wordt dan gevraagd of u blijft meedoen.

10. Welke informatie wordt er gevraagd te delen?
Om het onderzoek goed uit te kunnen voeren nemen we contact op met de huisarts, tandarts en thuiszorgorganisatie om de volgende informatie op te vragen over de proefpersoon *nadat* hij/zij hier schriftelijk toestemming voor heeft gegeven: ziekte geschiedenis, diagnoses en medicatiegebruik. Deze gegevens worden gecodeerd verwerkt. De naam en andere persoonsgegevens worden weggelaten. Alleen de onderzoekers van het onderzoeksteam weten welke code een proefpersoon heeft.

11. Wat gebeurt er met uw gegevens?
Alle gegevens worden verwerkt en 15 jaar opgeslagen onder een onderzoeksnummer. Alleen de onderzoekers van het onderzoeksteam weten welke code u heeft. De gegevens kunnen gecodeerd voor nader onderzoek op gebied van mondzorg en ouderenzorg worden, gebruikt zowel binnen als buiten Europa.
Daarnaast kan er om te controleren of het onderzoek goed en betrouwbaar uitgevoerd is een monitor die voor het Academisch Centrum Tandheelkunde Amsterdam (ACTA) werkt en de Inspectie voor de Gezondheidszorg uw persoonsgegevens inzien. Algemene informatie hierover vindt u in de brochure ‘Medisch-wetenschappelijk onderzoek’, bijlage D.

12. Zijn er extra kosten/is er een vergoeding wanneer u besluit aan dit onderzoek mee te doen?
Nee, er zijn geen kosten en/of vergoedingen verbonden aan deelname aan dit onderzoek.

13. Welke medisch-ethische toetsingscommissie heeft dit onderzoek goedgekeurd?
De Medisch Ethische Toetsingscommissie van het VU Medisch Centrum (METc VUmc) heeft dit onderzoek goedgekeurd. Meer informatie over de goedkeuring vindt u in de brochure ‘Medisch-wetenschappelijk onderzoek’, bijlage D.

14. Wilt u verder nog iets weten?
Bij vragen kunt u contact opnemen met de projectgroep ‘De mond niet vergeten!’ of dr. Weijenberg, contactgegevens kunt u vinden in bijlage **A**.
Wilt u graag een onafhankelijk advies over meedoen aan dit onderzoek? Dan kunt u terecht bij een onafhankelijk deskundige. Zijn gegevens vindt u in bijlage **A**.
Bij klachten kunt u het beste terecht bij de klachtenfunctionaris van de VU en ACTA.
Alle contactgegevens vindt u in bijlage **A**.

15. Ondertekening informed consent
Na één week bedenktijd, wordt u gevraagd over deelname aan het implementatieproject ‘De mond niet vergeten!’ en dit onderzoek. Indien u toestemming geeft, zouden wij u graag willen vragen;

- het toestemmingformulier

te ondertekenen bij een bijeenkomst van de implementatieproject ‘De mond niet vergeten!’.
Met het tekenen geeft u aan dat u de informatie heeft begrepen en instemt met deelname aan het onderzoek.

Het toestemmingsformulier wordt door de tandarts-onderzoeker, mw. Ho bewaard. U krijgt een kopie of een tweede exemplaar van de toestemmingsformulier.

Met vriendelijke groeten,

Mw. Bach Van Ho, tandarts-onderzoeker
dr. Roxane Weijenberg, onderzoeker
prof. dr. Frank Lobbezoo, supervisor

Bijlagen: **A** contactgegevens, **B** informatie over de verzekering, **C** toestemmingsformulier onderzoek zorgverlener, en **D** brochure ‘Medisch wetenschappelijk onderzoek.’

Bijlage A: contactgegevens

Projectgroep	De mond niet vergeten!
Correspondentie adres:	[redacted]
E-mail:	[redacted]

Onderzoeker:	Mw. Bach Van Ho, tandarts-onderzoeker
Correspondentie adres:	[redacted]
E-mail:	[redacted]
Telefoonnummer:	[redacted]
Telefonisch bereikbaar:	[redacted]

2de aanspreekpunt:	dr. Roxane Weijenberg, onderzoeker
Correspondentie adres:	[redacted]
E-mail:	[redacted]
Telefoonnummer:	[redacted]
Telefonisch bereikbaar:	[redacted]

Onafhankelijk deskundige:	dr. Peter Wetselaar, tandarts-onderzoeker
Correspondentie adres:	[redacted]
E-mail:	[redacted]
Telefoonnummer:	[redacted]
Telefonisch bereikbaar:	[redacted]

Bij afwezigheid kunt u contact op nemen met het Secretariaat van afdeling Orale Kinesiologie	
Secretaresse:	[redacted]
E-mail:	[redacted]
Telefoonnummer:	[redacted]
Telefonisch bereikbaar:	[redacted]

Klachten:	Klachtenfunctionaris van de VU en ACTA
Correspondentieadres:	[redacted]
Telefoonnummer:	[redacted]
Telefonisch beschikbaar:	[redacted]
Website met klachtenformulier:	[redacted]

Bijlage B: informatie over de verzekering

Voor iedereen die meedoet aan dit onderzoek, heeft het Academisch Centrum Tandheelkunde Amsterdam (ACTA) een verzekering afgesloten. De verzekering dekt schade door deelname aan het onderzoek. Dit geldt voor schade tijdens het onderzoek of binnen vier jaar na het einde ervan. Schade moet u binnen die vier jaar aan de verzekeraar hebben gemeld.

De verzekering dekt niet alle schade. Onderaan deze tekst staat in het kort welke schade niet wordt gedekt.
Deze bepalingen staan in het Besluit verplichte verzekering bij medisch-wetenschappelijk onderzoek met mensen. Dit besluit staat op www.ccmo.nl, de website van de Centrale Commissie Mensgebonden Onderzoek (zie 'Bibliotheek' en dan 'Wet- en regelgeving').

Bij schade kunt u direct contact leggen met de verzekeraar.
Dit is mogelijk via de website van de verzekeringsmaatschappij [redacted], u kunt natuurlijk ook een brief sturen naar onderstaande adres. In beide gevallen dient u het onderstaand polisnummer van de ACTA te vermelden.

De verzekeraar van het onderzoek is:	
Naam:	[redacted]
Adres:	[redacted]
Telefoonnummer:	[redacted]
Polisnummer:	[redacted]

De verzekering biedt een dekking van € 650.000 per proefpersoon maximaal en €5.000.000 voor het hele onderzoek en € 7.500.000 per jaar voor alle onderzoeken van dezelfde opdrachtgever.

De verzekering dekt de volgende schade **niet**:

- schade door een risico waarover u in de schriftelijke informatie bent ingelicht. Dit geldt niet als het risico zich ernstiger voordoet dan was voorzien of als het risico heel onwaarschijnlijk was;
- schade aan uw gezondheid die ook zou zijn ontstaan als u niet aan het onderzoek had meegedaan;
- schade door het niet (volledig) opvolgen van aanwijzingen of instructies;
- schade aan uw nakomelingen, als gevolg van een negatief effect van het onderzoek op u of uw nakomelingen;
- schade door een bestaande behandelmethode bij onderzoek naar bestaande behandelmethoden.

Bijlage C: toestemmingsformulier onderzoek zorgverlener

Toestemmingsformulier Het implementatieproject 'De mond niet vergeten!'
Voor de zorgverlener

Ik heb de informatiebrief voor de zorgverlener gelezen. Ik kon aanvullende vragen stellen. Mijn vragen zijn genoeg beantwoord. Ik had genoeg tijd om te beslissen of ik meedoe.

Ik weet dat meedoen helemaal vrijwillig is. Ik weet dat ik op ieder moment kan beslissen om toch niet mee te doen. Daarvoor hoef ik geen reden te geven.

Ik weet dat sommige mensen mijn gegevens kunnen zien. Die mensen staan vermeld in de informatiebrief.

Ik geef toestemming om mijn gegevens te gebruiken, voor de doelen die in de informatiebrief staan.

Ik geef toestemming om mijn onderzoeksgegevens 15 jaar na afloop van dit onderzoek te bewaren.

Ik geef toestemming om mijn gegevens gecodeerd over te dragen naar landen buiten de Europese Unie waar de Europese richtlijnen voor de bescherming van persoonsgegevens niet gelden. Dit gebeurt alleen bij noodzakelijkheid voor het onderzoek en nader onderzoek op gebied van mondzorg en ouderenzorg. De gegevens moeten gecodeerd worden overgedragen en zonder mijn naam.

Ik geef □ **wel**
□ **geen** toestemming om mij na dit onderzoek opnieuw te benaderen voor een vervolgonderzoek.

Ik wil meedoen aan dit onderzoek.

Naam zorgverlener:
Handtekening: Datum : __ / __ / __

(Dit gedeelte zal de onderzoeker ondertekenen)

Ik verklaar hierbij dat ik deze zorgverlener volledig heb geïnformeerd over het genoemde onderzoek.

Als er tijdens het onderzoek informatie bekend wordt die de toestemming van de zorgverlener zou kunnen beïnvloeden, dan breng ik hem/haar daarvan tijdig op de hoogte.

Naam onderzoeker (of diens vertegenwoordiger):
Handtekening: Datum: __ / __ / __

Aanvullende informatie is gegeven door (indien van toepassing):
Naam:
Functie:
Handtekening: Datum: __ / __ / __

Ministerie van Volksgezondheid,
Welzijn en Sport

Medisch-wetenschappelijk onderzoek

Algemene informatie voor de proefpersoon

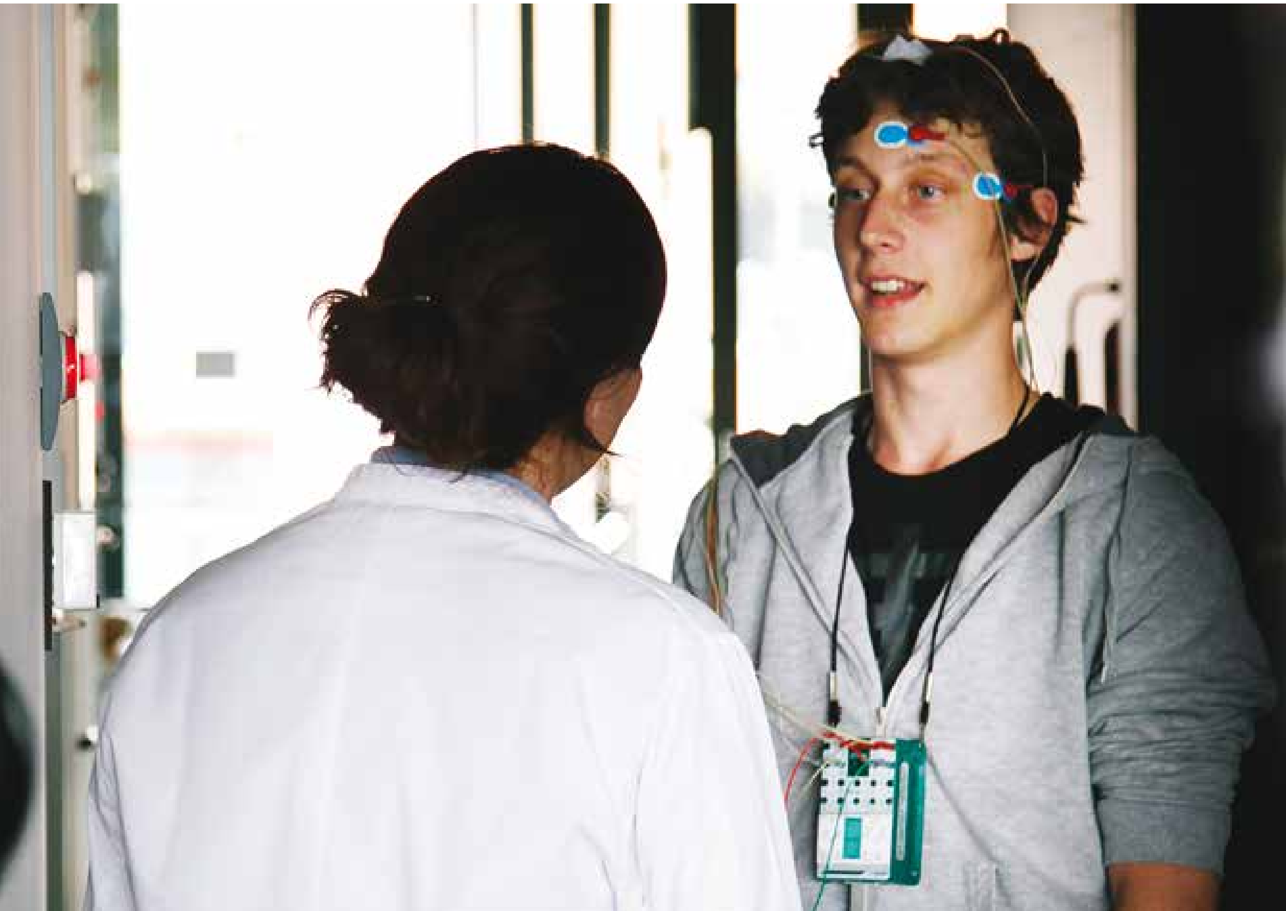

Inhoud

Inleiding 3

Medisch-wetenschappelijk onderzoek 4
- Wat is medisch-wetenschappelijk onderzoek?
- Wat zijn proefpersonen?
- Hoe gaat het onderzoek?

Meedoen 6
- Beslissing om mee te doen
- Wat levert medisch-wetenschappelijk onderzoek u op?
- Om rekening mee te houden
- Wat er gebeurt als u wel wilt meedoen
- Wat er gebeurt als u niet wilt meedoen
- Het kind als proefpersoon
- De wilsonbekwame proefpersoon

Rechten en plichten 12
- Uw rechten als proefpersoon
- Uw plichten als proefpersoon

Controle 14
- Wie controleert of het onderzoek goed gaat?
- Wat gebeurt er als iets misgaat?

Meer informatie 15
- Waar vindt u meer informatie?
- Waar kunt u terecht met klachten?

Bijlage 1: Vragenlijst voor de proefpersoon 17

Bijlage 2: De ontwikkeling van een nieuw medicijn 18

Bijlage 3: Ruimte voor eigen vragen en aantekeningen 19

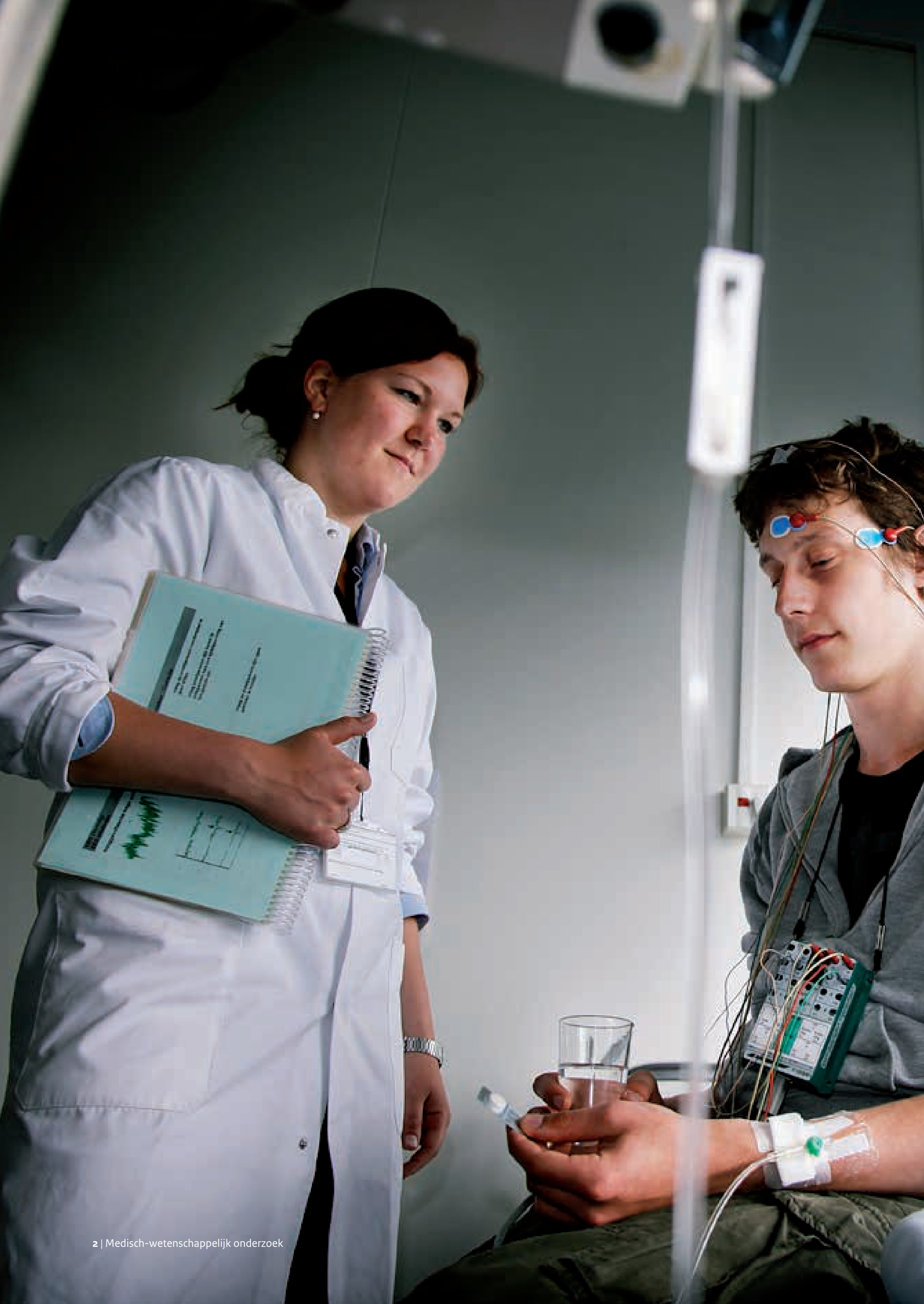

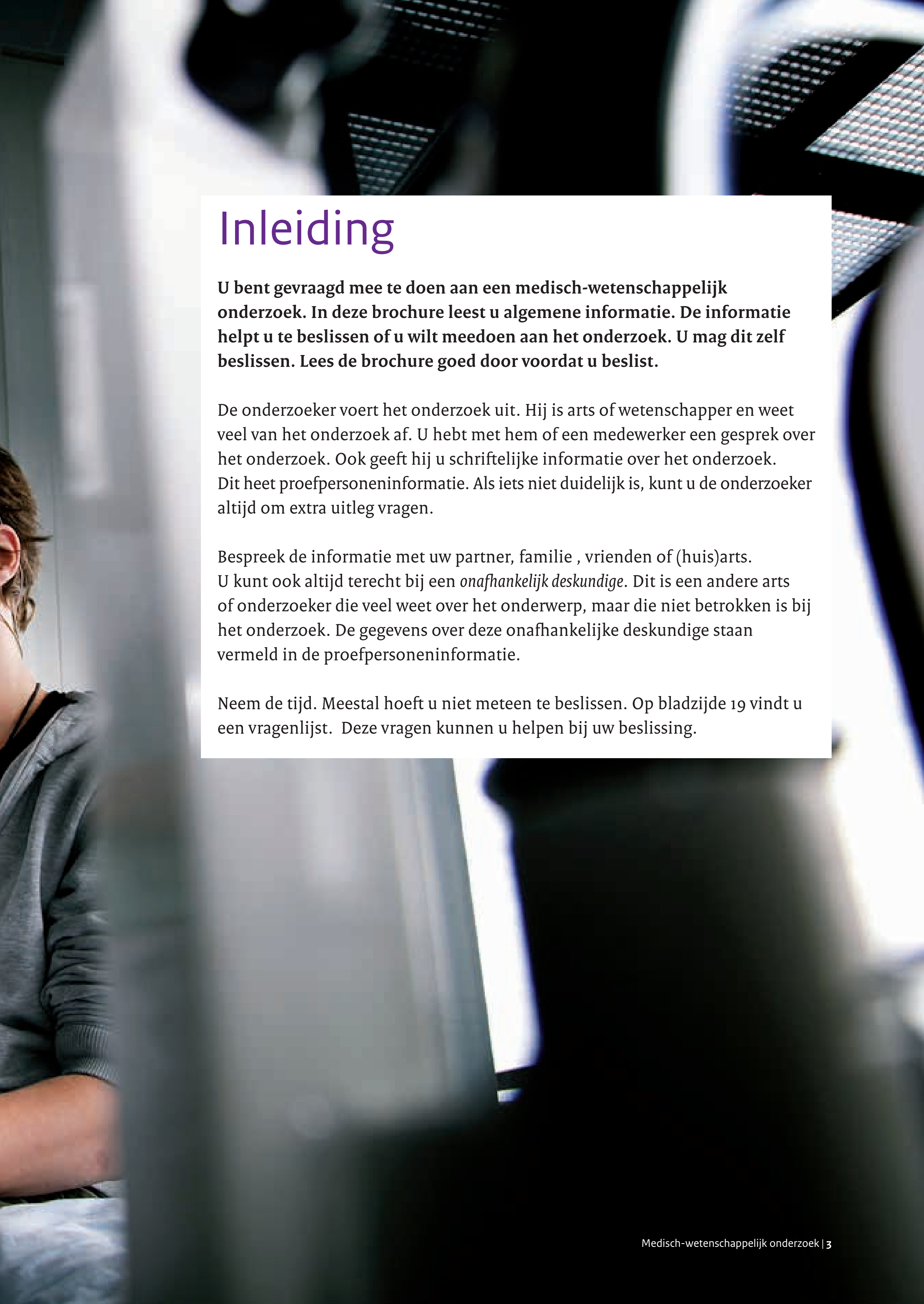

Inleiding

U bent gevraagd mee te doen aan een medisch-wetenschappelijk onderzoek. In deze brochure leest u algemene informatie. De informatie helpt u te beslissen of u wilt meedoen aan het onderzoek. U mag dit zelf beslissen. Lees de brochure goed door voordat u beslist.

De onderzoeker voert het onderzoek uit. Hij is arts of wetenschapper en weet veel van het onderzoek af. U hebt met hem of een medewerker een gesprek over het onderzoek. Ook geeft hij u schriftelijke informatie over het onderzoek. Dit heet proefpersoneninformatie. Als iets niet duidelijk is, kunt u de onderzoeker altijd om extra uitleg vragen.

Bespreek de informatie met uw partner, familie , vrienden of (huis)arts. U kunt ook altijd terecht bij een *onafhankelijk deskundige*. Dit is een andere arts of onderzoeker die veel weet over het onderwerp, maar die niet betrokken is bij het onderzoek. De gegevens over deze onafhankelijke deskundige staan vermeld in de proefpersoneninformatie.

Neem de tijd. Meestal hoeft u niet meteen te beslissen. Op bladzijde 19 vindt u een vragenlijst. Deze vragen kunnen u helpen bij uw beslissing.

Medisch-wetenschappelijk onderzoek

Wat is medisch-wetenschappelijk onderzoek?

Er zijn twee soorten medisch onderzoek. Als u gezondheidsklachten hebt, gaat u 'voor onderzoek' naar een arts. Die onderzoekt u om de oorzaak van uw klachten te vinden. Dit heet *diagnostisch* onderzoek. Het doel is om u beter te maken.
Er is ook *medisch-wetenschappelijk* onderzoek. Dit gebeurt niet in de eerste plaats om u beter te maken. De mensen die eraan meedoen heten proefpersonen.
Er zijn drie soorten medisch-wetenschappelijk onderzoek:

1. **Onderzoek om betere behandelingen voor ziekten te vinden.**
   Voorbeelden: een beter medicijn tegen hoofdpijn, een nieuwe soort hartklep of een nieuwe behandeling bij depressie

2. **Onderzoek om meer over een ziekte of de werking van het lichaam te weten te komen.**
   Voorbeelden: hoe reageert de huid op zonlicht? Krijg je van drop eten een hoge bloeddruk? Wat is de oorzaak van ADHD?

3. **Onderzoek om betere manieren te vinden voor het opsporen of het vaststellen van een ziekte.**
   Voorbeelden: hoe kunnen we (borst)kanker eerder opsporen? Hoe stellen we vast dat iemand een hartziekte heeft?

Wat zijn proefpersonen?

Iedereen die meedoet aan medisch-wetenschappelijk onderzoek, heet een proefpersoon. Er zijn twee soorten proefpersonen: gezonde vrijwilligers en patiënten. Patiënten kunnen soms voordeel hebben van het onderzoek.

Hoe gaat het onderzoek?

In veel onderzoeken worden alleen gegevens verzameld. Bijvoorbeeld over de lichaamstemperatuur, bepaalde bloedwaarden of hoe u zich voelt. U werkt dan mee aan bijvoorbeeld een lichamelijk onderzoek, een test of meting. Soms moet u zelf een lijstje bijhouden.

Er is ook onderzoek waarbij een nieuwe behandeling, operatie of medicijn wordt getest. We gebruiken voor het gemak hier het woord 'behandeling'.

Meestal vergelijkt de onderzoeker die nieuwe behandeling met de bestaande. Door loting bepaalt de onderzoeker wie welke behandeling krijgt. Dit heet randomisatie. Het hangt van het toeval af in welke groep u terechtkomt.
Vaak weet de onderzoeker zelf ook niet in welke groep u zit. Dat onderzoek heet dan dubbelblind: u en de onderzoeker weten allebei niet in welke groep u zit. Zo kan de onderzoeker de resultaten tussen de groepen eerlijker vergelijken.

Soms vergelijkt de onderzoeker een nieuwe behandeling met een ‘nepbehandeling’. Zo’n nepbehandeling heet een placebo. De placebo lijkt in alles op de nieuwe behandeling. De ene groep proefpersonen krijgt dus de nieuwe behandeling en de andere groep proefpersonen de nepbehandeling: de placebo.

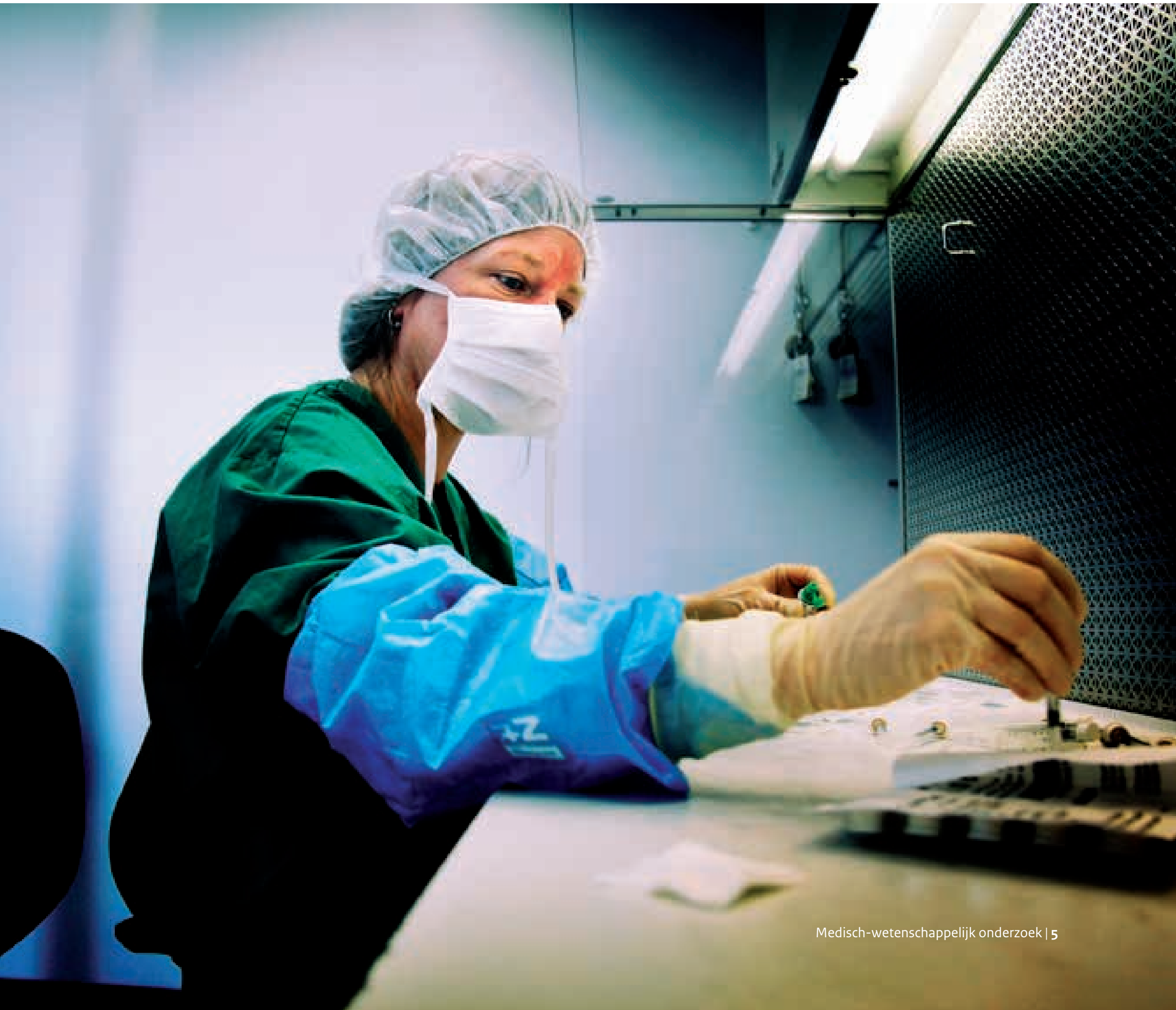

Meedoen

Beslissing om mee te doen
U beslist altijd zelf of u meedoet aan een onderzoek. Meedoen is *vrijwillig*. U bent dus nooit verplicht om mee te doen. Beslis daarom pas als u genoeg weet over de inhoud, eventuele risico's en voordelen van het onderzoek.

Wat levert medisch-wetenschappelijk onderzoek u op?
- U helpt mee aan de vooruitgang van de wetenschap.
- Bent u patiënt? Dan kunt u voordeel hebben van een nieuwe behandeling. Maar soms ook niet. Het onderzoek kan bijvoorbeeld ook bijdragen aan kennis over het ziekteproces. De onderzoeker zal u hier meer over vertellen. U verdient meestal geen geld met het onderzoek. U krijgt vaak wel de reiskosten vergoed.
- Bent u een gezonde vrijwilliger? Dan krijgt u vaak een financiële vergoeding. Meestal moet u hier wel nog zelf belasting over betalen. Reiskosten krijgt u bijna altijd vergoed.

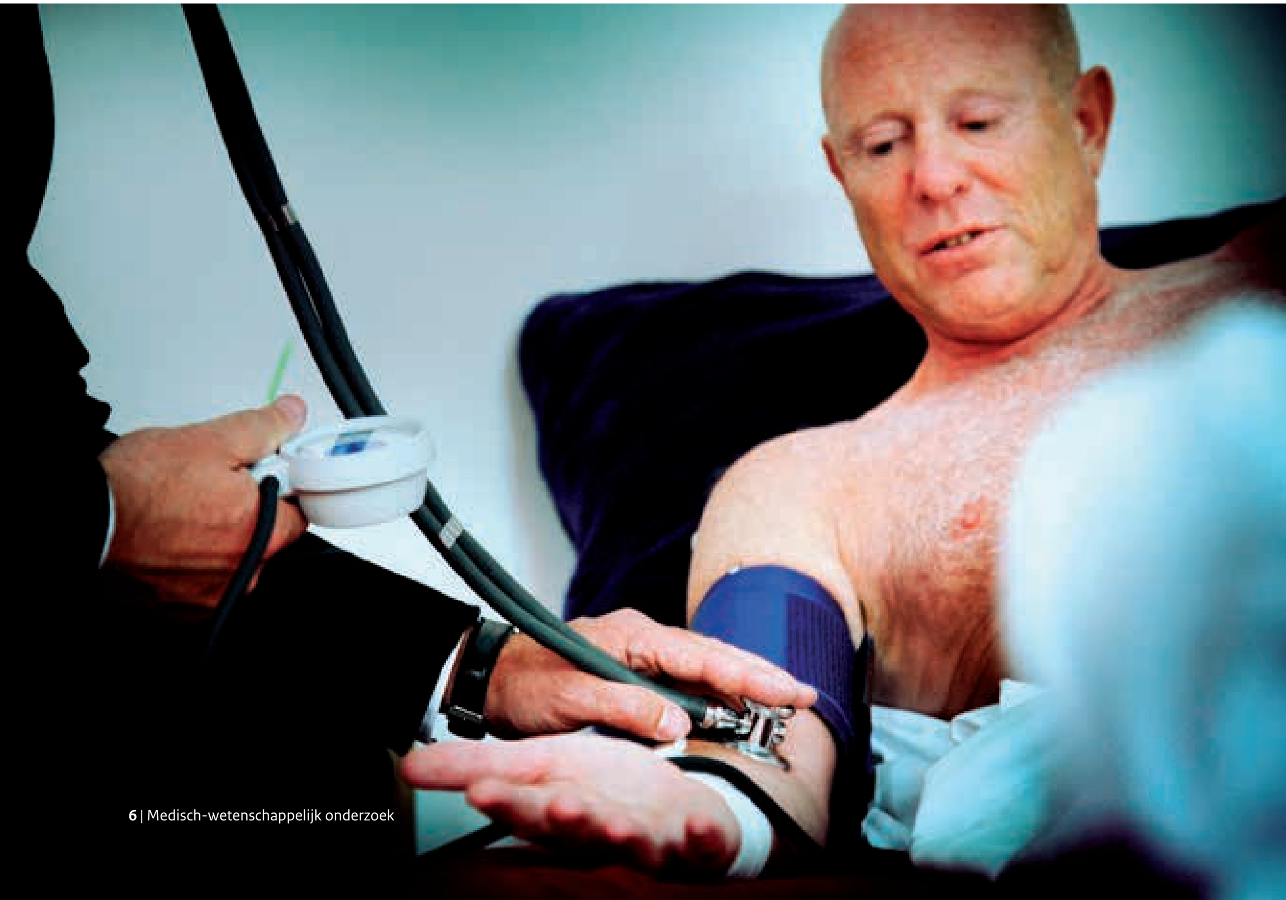

Om rekening mee te houden

- Bent u patiënt? Als u meedoet, moet u meestal **vaker op controle komen**. Soms moet u een lijstje bijhouden. Meedoen kost soms dus extra inspanning en tijd.
- Bent u een gezonde vrijwilliger? Ook dan **kost** meedoen **tijd**. Bijvoorbeeld een halve dag, of een aantal korte bezoeken. Maar soms wordt u ook een paar dagen opgenomen.
- U loopt soms een **risico** als u meedoet aan onderzoek. Want de behandeling is nieuw en wordt nog onderzocht. Niet alle effecten en bijwerkingen zijn al bekend. Ook doet de onderzoeker vaak extra testen en neemt bloed af. Hoe groot het risico is, hangt af van het soort onderzoek en van uw gezondheid. De onderzoeker legt u dit uit.
- Meedoen aan onderzoek kan **zwaar of vervelend zijn**, want:
  - soms is extra lichamelijk onderzoek nodig
  - soms krijgt u vragen over nare ervaringen
  - soms moet u stoppen met de medicijnen die u zelf al gebruikt
  - soms zijn er speciale leefregels, bijvoorbeeld voor alcohol, roken of het gebruik van voorbehoedsmiddelen.

Wat er gebeurt als u *wel* wilt meedoen

Als u wilt meedoen, ondertekent u een verklaring. Dit heet het toestemmingsformulier. Met uw handtekening zegt u dat u vrijwillig meewerkt aan het onderzoek. U krijgt zelf ook een exemplaar.

Vaak wordt u voor het onderzoek gekeurd. De onderzoeker kijkt dan of u lichamelijk geschikt bent. Het kan zijn dat u uiteindelijk toch niet kunt meedoen. U hoort dit van de onderzoeker.

Bedenkt u zich? U hebt altijd het recht om toch niet mee te doen. Ook tijdens het onderzoek mag u altijd stoppen. U hoeft niet te vertellen waarom. Bent u patiënt? Dan krijgt u gewoon de behandeling die u anders ook zou krijgen.

Wat er gebeurt als u *niet* wilt meedoen

Als u niet wilt meedoen, hoeft u verder niets te doen. U hoeft niets te tekenen. U hoeft ook niet te vertellen waarom u niet wilt meedoen. Bent u patiënt? Dan krijgt u gewoon de behandeling die u anders ook zou krijgen.

Het kind als proefpersoon

Ook kinderen en jongeren onder de 18 jaar kunnen deelnemen aan medisch-wetenschappelijk onderzoek. In deze brochure wordt voor het gemak het woord 'kind' gebruikt voor iedereen onder de 18 jaar.

Medisch-wetenschappelijk onderzoek met kinderen mag alleen als het aan strenge regels voldoet. Deze regels gaan bijvoorbeeld over de risico's waaraan het kind als proefpersoon mag worden blootgesteld. Die risico's mogen niet te groot zijn. Daarnaast gelden er aparte regels voor de toestemming. Deze staan hieronder vermeld.

Is uw kind gevraagd om mee te doen aan onderzoek? *

Wil uw kind meedoen aan het onderzoek? En stemt u ermee in dat uw kind meedoet? Dan tekent u een toestemmingsformulier. Dit moet altijd getekend worden door beide ouders (of voogd). Is uw kind tussen de 12 en 18 jaar, dan moet het zelf ook een handtekening zetten op een toestemmingsformulier. Pas dan mag het kind meedoen aan onderzoek.

Stoppen

Bedenkt u zich? Uw kind heeft altijd het recht om toch niet mee te doen. Ook tijdens het onderzoek mag u (of uw kind) altijd besluiten te stoppen. U hoeft niet te vertellen waarom. Is uw kind patiënt, dan krijgt het gewoon de behandeling die het anders ook zou krijgen.

Als uw kind zich tijdens het onderzoek verzet - denk hierbij aan sterke angst, verdriet of boosheid - dan mag uw kind niet langer meedoen. Voordat het onderzoek start, overlegt de onderzoeker met u wat als verzet wordt gezien.

Wat gebeurt er als u niet akkoord bent met deelname van uw kind?

Wilt u niet dat uw kind meedoet aan het onderzoek? Dan hoeft u verder niets te doen. U hoeft niets te tekenen. U hoeft ook niet te vertellen waarom u niet wilt dat uw kind meedoet. Is uw kind patiënt? Dan krijgt het gewoon de behandeling die het anders ook zou krijgen.

* Bij het ter perse gaan van deze brochure staat de leeftijdsgrens waarbij ouders (of voogd) moeten instemmen met deelname van hun kind op 18 jaar. Het is mogelijk dat, op het moment dat u deze brochure leest, de wet is aangepast en er boven de 16 jaar geen toestemming van de ouders meer nodig is.

Ben je tussen de 12 en 18 jaar? En wil je meedoen aan onderzoek? *
Stemmen jouw ouders ermee in dat je meedoet? Dan teken je een toestemmingsformulier. Jouw ouders (of voogd) zetten ook hun handtekening op een toestemmingsformulier. Pas dan mag je meedoen aan het onderzoek.

Stoppen
Bedenk je je? Je hebt altijd het recht om toch niet mee te doen. Ook tijdens het onderzoek mag je altijd stoppen. Je hoeft niet te vertellen waarom. Ben je patiënt? Dan krijg je de normale behandeling die je anders ook zou krijgen.

Wat gebeurt er als je niet wilt meedoen?
Wil je niet meedoen aan het onderzoek? Dan hoef je verder niets te doen. Je hoeft niets te tekenen. Je hoeft ook niet te vertellen waarom je niet wil meedoen. Ben je patiënt? Dan krijg je de normale behandeling die je anders ook zou krijgen.

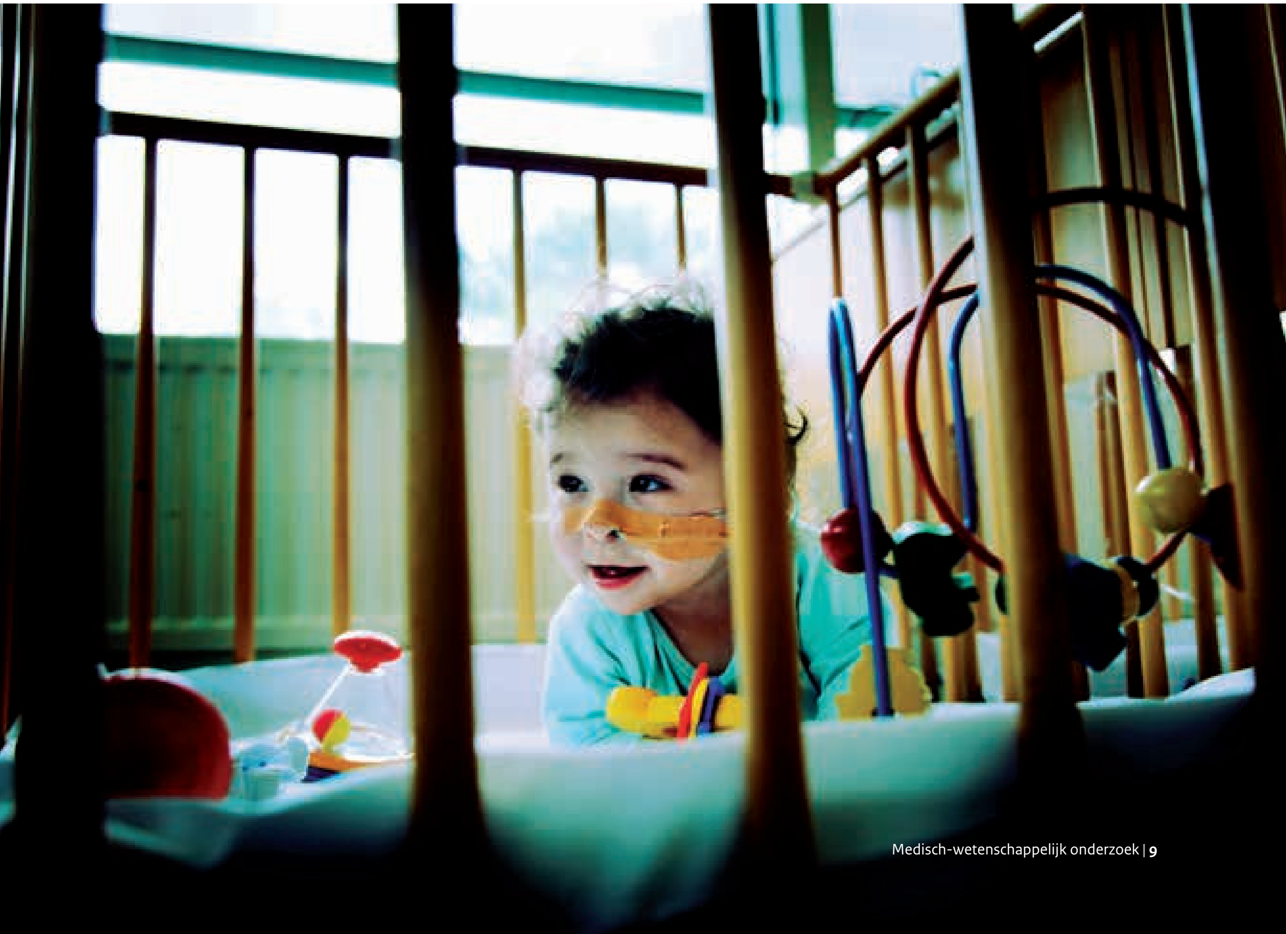

De wilsonbekwame proefpersoon

Zowel volwassenen (alle mensen van 18 jaar en ouder) als kinderen kunnen wilsonbekwaam zijn. Het gaat bijvoorbeeld om ouderen met dementie, verstandelijk gehandicapten, comapatiënten of mensen met een ernstige psychische ziekte. Zij kunnen hun eigen belang niet altijd goed beoordelen. Voor wilsonbekwame personen worden belangrijke beslissingen door een vertegenwoordiger genomen. Dit kunnen de ouders, echtgenoot of kinderen van de wilsonbekwame zijn. Soms is iemand door de rechter hiervoor aangewezen.

Onderzoek met wilsonbekwame proefpersonen mag alleen als het aan strenge regels voldoet. Deze regels gaan bijvoorbeeld over de risico's waaraan de wilsonbekwame proefpersoon mag worden blootgesteld. Die risico's mogen niet te groot zijn. Daarnaast gelden er aparte regels voor de toestemming. Deze staan hieronder vermeld.

Bent u vertegenwoordiger van een wilsonbekwame persoon?
Wil de onderzoeker dat de wilsonbekwame persoon meedoet aan een onderzoek? Als u ermee instemt dat deze meedoet, tekent u een toestemmingsformulier.

Stoppen
Bedenkt u zich? Ook tijdens het onderzoek mag u altijd besluiten dat de wilsonbekwame proefpersoon niet meer meedoet. U hoeft niet te vertellen waarom. Is de wilsonbekwame een patiënt, dan krijgt hij gewoon de behandeling die hij anders ook zou krijgen.
Als de wilsonbekwame proefpersoon zich tijdens het onderzoek verzet - denk hierbij aan sterke angst, verdriet of boosheid - mag deze niet langer meedoen. Voor het onderzoek start, overlegt de onderzoeker met u wat als verzet wordt gezien.

Wat gebeurt er als u niet akkoord bent met deelname van de wilsonbekwame persoon?
Wilt u niet dat de wilsonbekwame persoon meedoet aan het onderzoek? Dan hoeft u verder niets te doen. U hoeft niets te tekenen. U hoeft ook niet te vertellen waarom u dit niet wilt.
Is de wilsonbekwame een patiënt? Dan krijgt hij gewoon de behandeling die hij anders ook zou krijgen.

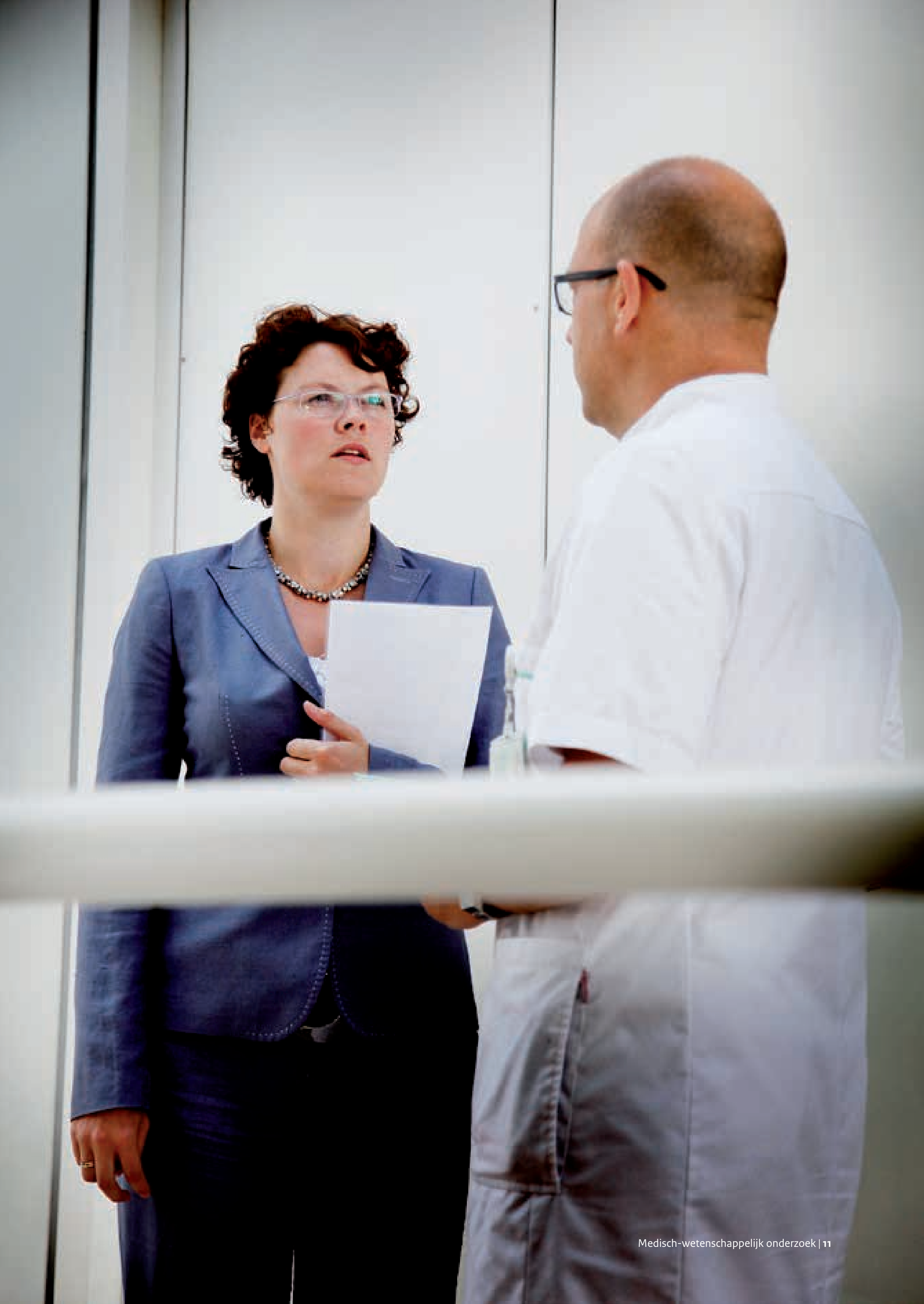

Rechten en plichten

Uw rechten als proefpersoon

Als proefpersoon hebt u rechten. Die zijn vastgelegd in de Wet medisch-wetenschappelijk onderzoek met mensen. De belangrijkste zijn:

Recht om zelf te beslissen

U beslist zelf of u meedoet aan een onderzoek. Het is uw eigen keuze.
Ook als uw arts u vraagt mee te doen, mag u altijd nee zeggen.

Recht op informatie en vragen stellen

De onderzoeker is verplicht vooraf een gesprek met u te voeren. Ook moet hij u schriftelijke informatie over het onderzoek geven. U mag altijd alle vragen stellen die bij u opkomen. Dat mag voor, tijdens en na het onderzoek. De onderzoeker moet uw vragen beantwoorden.
Er is ook een onafhankelijk deskundige. Die weet veel over het onderwerp, maar is niet betrokken bij het onderzoek. Aan hem kunt u ook vragen stellen over het onderzoek. Dit kan meestal telefonisch.

Recht op bedenktijd

Meestal hoeft u niet meteen te beslissen of u aan het onderzoek meedoet.
U hebt het recht om de informatie (thuis) rustig te bekijken. Soms moet u wel snel beslissen, bijvoorbeeld op de spoedeisende hulp.

Recht te stoppen met het onderzoek

U mag altijd zeggen dat u toch niet meedoet. Ook als het onderzoek al is begonnen.
U hoeft niet te vertellen waarom. Als u stopt, heeft dat geen invloed op de behandeling die u voor het onderzoek kreeg. Soms kunt u niet direct stoppen, omdat dat slecht kan zijn voor uw gezondheid. Vertel de onderzoeker daarom altijd dat u wilt stoppen.

Recht op bescherming van uw gegevens

Tijdens het onderzoek verzamelt de onderzoeker gegevens over u. Deze gegevens blijven geheim en krijgen een code. Daarom komt u uw naam nooit tegen in een rapport over het onderzoek. In rapporten over het onderzoek wordt die code gebruikt. Behalve de onderzoeker kunnen ook andere mensen uw gegevens zien. In het toestemmingsformulier of de proefpersoneninformatie staat aangegeven welke mensen dat zijn. Deze mensen controleren of het onderzoek goed en betrouwbaar is. Denk hierbij aan het onderzoeksteam en de Inspectie voor de Gezondheidszorg (IGZ). In onder andere de Wet bescherming persoonsgegevens (Wbp) is vastgelegd hoe dit moet.

De onderzoeker bewaart uw gegevens tijdens het onderzoek. U geeft alleen toestemming voor gebruik van uw gegevens voor dit onderzoek.
U kunt ook toestemming geven voor gebruik van uw gegevens voor later onderzoek. De onderzoeker bewaart dan uw gegevens. Als hij die gegevens dan later wil gebruiken voor een nieuw onderzoek, vraagt hij u opnieuw om toestemming. Hierover staat meer in de proefpersoneninformatie die u hebt gekregen van de onderzoeker.

Lichaamsmateriaal

Mogelijk wordt bij u bloed, speeksel of ander lichaamsmateriaal afgenomen.
Dit mag enkel gebruikt worden voor het onderzoek waar u toestemming voor hebt gegeven. Het kan zijn dat de onderzoeker het lichaamsmateriaal later ook wil gebruiken voor een ander onderzoek. Mocht hier sprake van zijn, dan moet de onderzoeker hier apart toestemming voor vragen. Hierover staat meer in de proefpersoneninformatie die u hebt gekregen van de onderzoeker.

Uw plichten als proefpersoon

U moet zich houden aan de regels van het onderzoek. Die regels zijn voor elk onderzoek verschillend. Soms moet u zonder dat u iets gegeten en gedronken hebt beginnen aan het onderzoek. Soms moet u elke dag op een vast tijdstip een pil innemen. Of bijvoorbeeld elke dag opschrijven hoe u zich voelt.

Het is belangrijk dat u zich aan deze regels houdt. Anders kan de onderzoeker het onderzoek niet goed uitvoeren en kloppen de uitkomsten niet meer.
De onderzoeker kan dan beslissen dat u niet meer kunt meedoen aan het onderzoek.

Controle

Wie controleert of het onderzoek goed gaat?

In Nederland zijn er strenge regels voor onderzoek met proefpersonen.
Die staan in de Wet medisch-wetenschappelijk onderzoek met mensen.

Een speciale commissie beoordeelt van tevoren elk onderzoek. Deze commissie heet de medisch-ethische toetsingscommissie. Hier gebruiken we voor het gemak enkel het woord 'toetsingscommissie'. Een onderzoek mag pas starten als de toetsingscommissie het heeft goedgekeurd. De toetsingscommissie beoordeelt onder andere:

- of het onderzoek nuttig is
- of het onderzoek goed is opgezet
- of alle informatie die u krijgt juist is
- of de risico's van het onderzoek niet te groot zijn
- of het onderzoek niet te veel van u vraagt.

De mensen in de toetsingscommissie hebben veel verstand van onderzoek.
Zij zijn bijvoorbeeld arts of kennen de wetten en regels heel goed. Zij hebben zelf geen belang bij het onderzoek. Wilt u weten welke commissie uw onderzoek heeft beoordeeld? Dat staat in de proefpersoneninformatie van de onderzoeker.

Wat gebeurt er als iets misgaat?

Onderzoekers willen natuurlijk niet dat er tijdens het onderzoek iets misgaat.
Maar er kan wel eens een probleem ontstaan. Daarom is er een verzekering voor proefpersonen afgesloten. Alleen bij onderzoek zonder risico's hoeft dat niet.
Hebt u schade door het wetenschappelijk onderzoek? Dan kunt u contact opnemen met de onderzoeker of met de verzekeraar van het onderzoek. Hierover staat meer in de proefpersoneninformatie die u hebt gekregen van de onderzoeker.

Meer informatie

Waar vindt u meer informatie?

- Bij de **onderzoeker**. U krijgt schriftelijke proefpersoneninformatie van de onderzoeker. Die gaat speciaal over het onderzoek waarvoor u bent gevraagd. Ook kunt u de onderzoeker altijd vragen stellen.
- Bij de **onafhankelijk deskundige**. U vindt zijn naam en telefoonnummer in de informatie die u van de onderzoeker krijgt.
- Op **www.ccmo.nl**. Dit is de website van de Centrale Commissie Mensgebonden Onderzoek, de CCMO. De CCMO houdt het wetenschappelijk onderzoek met mensen goed in de gaten. Op deze website vindt u algemene informatie.

Waar kunt u terecht met klachten?

Het onderzoek mag niet betekenen dat u aan onnodige (vermijdbare) risico's wordt blootgesteld. Klachten over het onderzoek kunt u het beste bespreken met de onderzoeker. Wilt u dit liever niet? Dan kunt u ook terecht bij de klachtencommissie van de instelling waar het onderzoek wordt gedaan. U vindt de telefoonnummers in de informatie van de onderzoeker.
Ook kunt u een melding doen bij de Inspectie voor de Gezondheidszorg, de IGZ (www.igz.nl). Op deze website staat wanneer de IGZ een melding onderzoekt en zo ja, hoe.

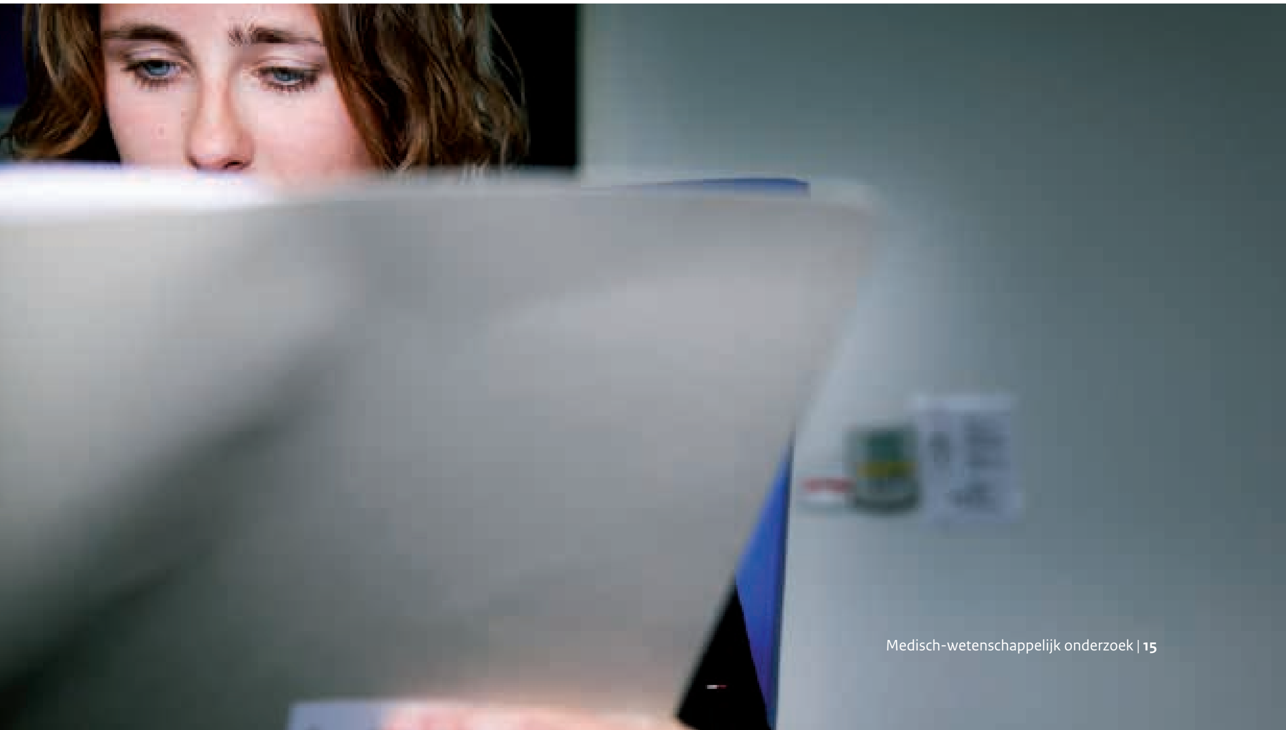

Tip
U mag iemand meenemen naar uw gesprek met de onderzoeker. Twee horen meer dan één. Ook kan het helpen om belangrijke dingen op te schrijven.

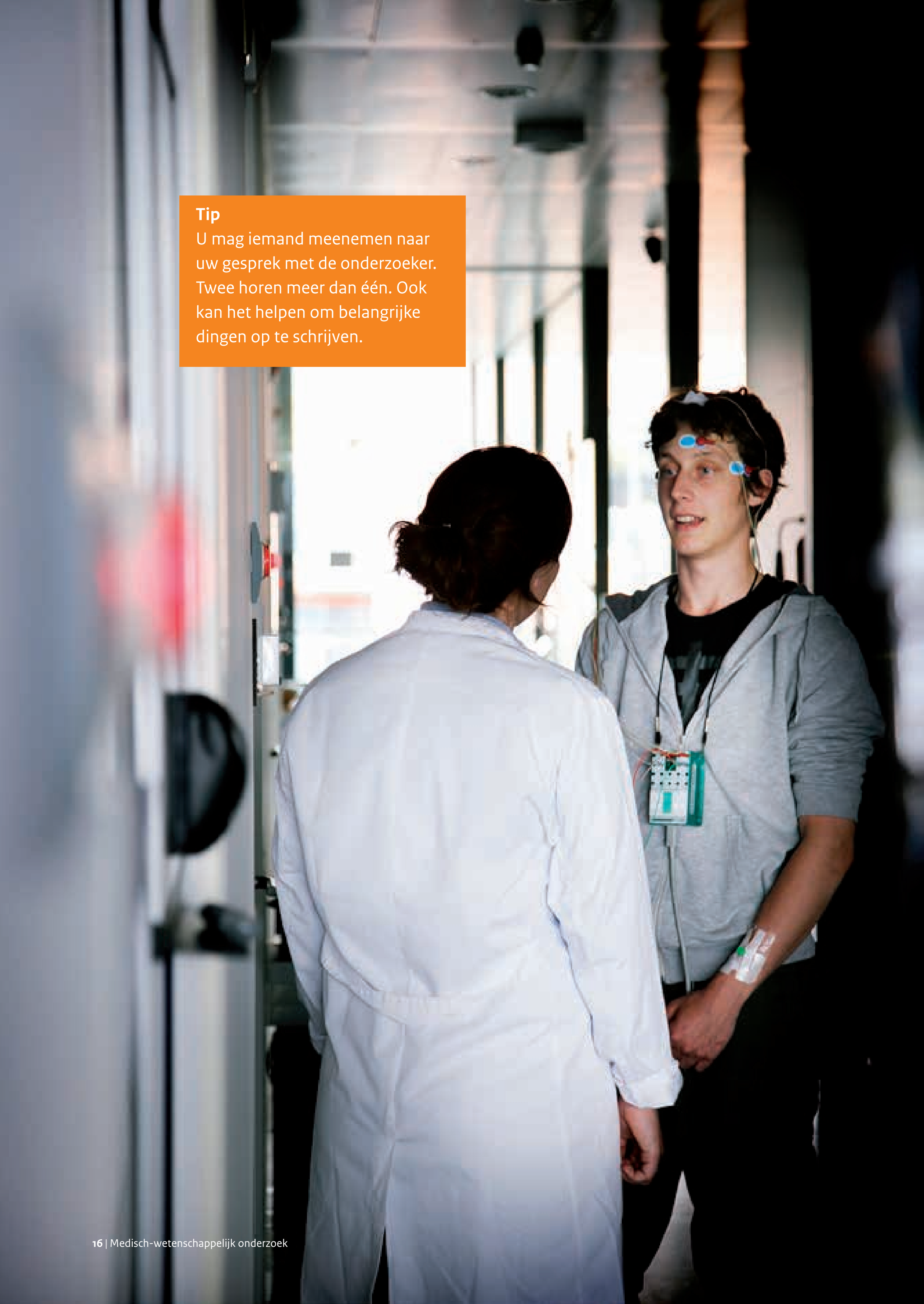

Bijlage 1: Vragenlijst voor de proefpersoon

Lees deze vragen voordat u beslist of u meedoet. De vragen kunnen u helpen bij uw beslissing. In de proefpersoneninformatie van de onderzoeker vindt u veel antwoorden. Deze informatie krijgt u van de onderzoeker. Hebt u vragen? Stel ze aan de onderzoeker.

1. Wat is het **doel** van het onderzoek?

2. Wat is het **nut** van het onderzoek voor mij?

3. **Waarom** vraagt de onderzoeker mij?

4. **Hoeveel tijd** kost het onderzoek mij?

5. Wat moet ik precies **doen en laten** voor het onderzoek?

6. Welke **risico's** of **bijwerkingen** zijn mogelijk?

7. Ik wil graag **zwanger** worden. Mag ik toch meedoen?

8. Moet ik **stoppen** met mijn **medicijnen**?

9. Bij wie kan ik terecht als er tijdens het onderzoek **iets misgaat**?

10. Is er een **proefpersonenverzekering** afgesloten voor het onderzoek?

11. Wat gebeurt er met mijn **gegevens**?

12. Krijg ik de **resultaten** van het onderzoek te horen?

13. Wanneer hoor ik **welke behandeling** ik heb gekregen?

14. Bij wie kan ik terecht met **vragen**?

Bijlage 2: De ontwikkeling van een nieuw medicijn

Ongeveer een derde van het medisch-wetenschappelijk onderzoek is geneesmiddelenonderzoek. Voordat patiënten een nieuw medicijn (geneesmiddel) krijgen, moeten onderzoekers weten of het medicijn veilig is. Een medicijn wordt in drie stappen ontwikkeld:

1. **Laboratorium**
   In het laboratorium zijn onderzoekers steeds op zoek naar nieuwe medicijnen. Daar wordt een nieuw medicijn uitgebreid getest.
2. **Proefdieren**
   Als de testen in het laboratorium positief uitvallen, volgt onderzoek bij proefdieren. De onderzoekers kijken welk effect het medicijn op dieren heeft. Ook kijken ze of er bijwerkingen zijn.
3. **Onderzoek bij mensen**
   Dierproeven laten zien of het medicijn veilig lijkt en resultaat heeft. Als dat zo is, begint pas het onderzoek bij mensen. Er is dus al veel onderzocht voordat u wordt gevraagd om mee te doen. Geneesmiddelenonderzoek bij mensen bestaat uit vier fasen:

   Fase I: Is het medicijn veilig?
   De onderzoekers kijken hoe proefpersonen (meestal gezonde vrijwilligers) het medicijn verdragen. Ook kijken de onderzoekers hoe het middel werkt in het lichaam.
   Fase II: Werkt het medicijn?
   Als het middel veilig genoeg is, wordt het bij een klein aantal patiënten getest. Nu kijken de onderzoekers of het ook echt werkt.
   Fase III: Werkt het beter dan bestaande medicijnen?
   Zijn de resultaten van fase II goed, dan vragen de onderzoekers meer patiënten om mee te doen. Ze vergelijken het nieuwe medicijn dan vaak met een bestaand medicijn. Is het resultaat goed? Dan volgt vaak registratie van het nieuwe medicijn als officieel geneesmiddel. Artsen mogen het dan voorschrijven.
   Fase IV: Wat zijn de gevolgen op lange termijn?
   Ook medicijnen die de artsen al voorschrijven, worden nog onderzocht. Dit kan bij tienduizenden patiënten gebeuren. Meestal gaat het dan om bijwerkingen op de lange termijn. Soms wordt onderzocht of een medicijn ook helpt tegen andere ziektes.

Bijlage 3: Ruimte voor eigen vragen en aantekeningen

Colofon
Deze brochure is tot stand gekomen in opdracht van het ministerie van Volksgezondheid, Welzijn en Sport in samenwerking met de Centrale Commissie Mensgebonden Onderzoek en de erkende medisch-ethische toetsingscommissies.

Voor informatie en vragen over bestellingen kunt u terecht bij Informatie Rijksoverheid:
1400 (lokaal tarief).
De medewerkers zijn bereikbaar op werkdagen van 08.00 tot 20.00 uur.

www.rijksoverheid.nl

Voor meerdere exemplaren
brochures@minvws.nl

Oplage: 250.000
September 2014
